# Deciphering the Role of Cancer Stem Cells: Drivers of Tumor Evolution, Therapeutic Resistance, and Precision Medicine Strategies

**DOI:** 10.3390/cancers17030382

**Published:** 2025-01-24

**Authors:** Mohamed El-Tanani, Syed Arman Rabbani, Shakta Mani Satyam, Imran Rashid Rangraze, Adil Farooq Wali, Yahia El-Tanani, Alaa A. A. Aljabali

**Affiliations:** 1 RAK College of Pharmacy, Ras Al Khaimah Medical and Health Sciences University, Ras Al Khaimah P.O. Box 11172, United Arab Emirates; 2 Department of Clinical Pharmacy, RAK College of Pharmacy, Ras Al Khaimah Medical and Health Sciences University, Ras Al Khaimah P.O. Box 11172, United Arab Emirates; 3 Department of Pharmacology, RAK College of Medical Sciences, Ras Al Khaimah Medical and Health Sciences University, Ras Al Khaimah P.O. Box 11172, United Arab Emirates; 4 Department of Internal Medicine, RAK College of Medical Sciences, Ras Al Khaimah Medical and Health Sciences University, Ras Al Khaimah P.O. Box 11172, United Arab Emirates; 5 Department of Medicinal Chemistry, RAK College of Pharmacy, Ras Al Khaimah Medical and Health Sciences University, Ras Al Khaimah P.O. Box 11172, United Arab Emirates; 6 Royal Cornwall Hospital NHS Trust, Truro TR1 3LJ, UK; 7 Department of Pharmaceutics and Pharmaceutical Technology, Faculty of Pharmacy, Yarmouk University, Irbid 21163, Jordan

**Keywords:** cancer stem cells, tumor heterogeneity, signaling pathways, tumor microenvironment, therapeutic resistance, artificial intelligence

## Abstract

Cancer stem cells (CSCs) play a central role in tumor progression, recurrence, and resistance to conventional therapies, making them a critical focus in oncology research. This review provides a comprehensive analysis of CSC biology, emphasizing their self-renewal, differentiation, and dynamic interactions with the tumor microenvironment (TME). Key signaling pathways, including Wnt, Notch, and Hedgehog, are discussed in detail to highlight their potential as therapeutic targets. Current methodologies for isolating CSCs are critically examined, addressing their advantages and limitations in advancing precision medicine. Emerging technologies, such as CRISPR/Cas9 and single-cell sequencing, are explored for their transformative potential in unraveling CSC heterogeneity and informing therapeutic strategies. The review also underscores the pivotal role of the TME in supporting CSC survival, promoting metastasis, and contributing to therapeutic resistance. Challenges arising from CSC-driven tumor heterogeneity and dormancy are analyzed, along with strategies to mitigate these barriers, including novel therapeutics and targeted approaches. Ethical considerations and the integration of artificial intelligence in designing CSC-specific therapies are discussed as essential elements of future research. The manuscript advocates for a multi-disciplinary approach that combines innovative technologies, advanced therapeutics, and collaborative research to address the complexities of CSCs. By bridging existing gaps in knowledge and fostering advancements in personalized medicine, this review aims to guide the development of more effective cancer treatment strategies, ultimately improving patient outcomes.

## Simple Summary

Cancer stem cells (CSCs) are pivotal in tumor growth, recurrence, and resistance to standard therapies. This review delves into CSCs’ ability to self-renew and differentiate, fueling tumor heterogeneity and metastasis. It highlights critical signaling pathways like Wnt, Notch, and Hedgehog, which are essential for targeting CSCs. Emerging technologies, such as CRISPR/Cas9 and single-cell sequencing, promise to enhance CSC research and management. The review also explores the complex interaction between CSCs and the tumor microenvironment (TME), which influences therapeutic effectiveness. Addressing CSC-driven resistance, the article proposes advanced strategies for overcoming these challenges. It emphasizes the role of artificial intelligence in designing personalized, CSC-targeted therapies, ushering in a new era for precision medicine in oncology. This comprehensive approach aims to improve cancer treatment outcomes through innovative technologies and inter-disciplinary collaboration.

## 1. Introduction

Cancer stem cells, also known as CSCs, are a class of cells that are found within a tumor which possess the characteristics of stem cells, including the ability to divide and form the different cell types that make up the tumor [1,2]. These cells possess a high rate of resistance and the capability to initiate and sustain tumor growth, comparable to the stem cells that are found in healthy tissues that are responsible for regeneration and repair [3]. Bonnet and Dick first discovered these cells in acute myeloid leukemia [4]. Following their discovery, several other researchers have isolated these cells in various solid tumors such as breast [5], brain [6], prostate [7], ovarian [8], and pancreatic [9] tumors, among others. With the discovery of CSCs, the understanding of tumor biology has revolutionized the fact that not all the cancer cells are equal in the development of the disease and its recurrence [10].

Furthermore, CSCs possess various characteristics, such as an unorthodox metabolic profile, enhanced efflux transporters for drugs, a highly efficient DNA repair system and epithelial-to-mesenchymal transition (EMT) [11,12]. Also, CSCs create a specific structure within the tissue called a niche, which is crucial for CSC functions [13]. These features are critical in regulating the proliferation and tumorigenic potential of CSCs. Also, CSCs’ role in cancer spread has also been proven [14]. Metastasis is a complex process in which CSCs detach from the primary tumor, survive and circulate within the bloodstream, and subsequently colonize distant organs, forming secondary tumors that are often more aggressive than the primary tumor.

CSCs’ studies have helped to reveal certain issues in conventional cancer treatment, including the issue of resistance to conventional drugs and the propensity of tumors to recur and spread [15,16]. Standard antimitotic drugs, like chemotherapy and radiation therapy, affect proliferating cells to a greater extent and may spare the quiescent CSCs, which are relatively more resistant to these forms of treatment [17]. CSCs are said to be resistant to these therapies, thereby leading to recurrence and the progression of tumors to other parts of the body even after a good initial response to treatment [18]. Hence, there is a need to develop new strategies in cancer treatment that will target these specific cells. To design new and more effective treatments that can prevent cancer relapse and improve patients’ outcomes, it is crucial to identify the characteristics of CSCs, their markers, and signaling pathways [19]. Strategies could involve inhibiting the pathways which are crucial for CSCs’ self-renewal and survival to make CSCs more sensitive to the standard therapies, or to induce the differentiation of CSCs into less dangerous cell types. Targeting CSCs effectively can lead to several benefits such as small tumor size and no cancer recurrence and spread, therefore providing a better and long-lasting treatment for patients.

The focus on CSCs in cancer research has revolutionized our understanding of tumor biology, challenging the traditional view that all cancer cells contribute equally to tumor development, progression, and recurrence. This paradigm shift offers hope for the development of targeted therapies that address the root cause of therapeutic resistance and tumor relapse. By deciphering the unique characteristics, signaling pathways, and metabolic profiles of CSCs, researchers aim to design precision medicine strategies that effectively target these cells. Such strategies hold the promise of reducing tumor size, preventing recurrence and metastasis, and ultimately, improving patient outcomes. Understanding and targeting CSCs is not just an advancement in cancer research; it is a pivotal step toward redefining cancer treatment in the era of precision medicine.

## 2. Characteristics of Cancer Stem Cells

**Figure 1:**
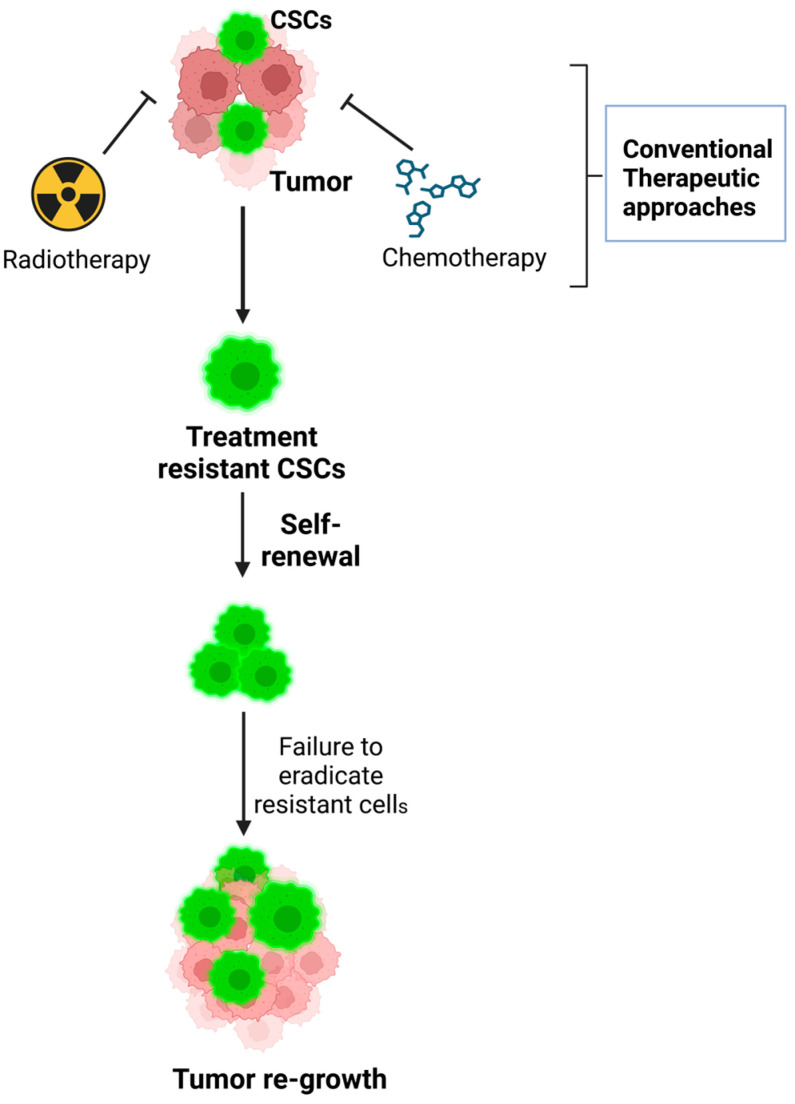
Characteristics of cancer stem cells (CSCs). CSCs are the malignant counterparts of normal somatic stem cells, possessing the capacities for self-renewal and differentiation. CSCs can remain in a quiescent state, under drug pressure, but are also capable of undergoing symmetric division to ensure self-renewal, thereby supporting tumor growth and propagation. Additionally, they can undergo asymmetric division, contributing to the regeneration and maintenance of the heterogeneous tumor cell population within the neoplasm.

Due to the presence of certain characteristics, CSCs can be distinguished from other cancer cells and are involved in the formation of tumors, their spread and treatment resistance. The features consist of self-renewal, differentiation and the ability to withstand conventional cancer therapy (Figure 1).

CSCs possess the property of self-renewal, whereby they are able to maintain their population in the tumor over time [20]. These cells can divide by symmetrical division to produce either two CSCs or one CSC and one non-CSC daughter cell [21]. Through symmetrical division, CSCs are able to proliferate to high levels, which results in cell growth and tumor formation and development [22]. When the CSCs isolated from the primary tumor of a patient were injected into mice with severe combined immunodeficiency disease, the CSCs were able to form new tumors [23].

Self-renewal, a hallmark capacity of CSCs, is regulated by specific signaling pathways, including Wnt [24], Notch [25], and Hedgehog [26]. These pathways, often dysregulated in cancer, represent a critical link to the tumorigenic potential of CSCs. Targeting these self-renewal pathways offers promising therapeutic strategies to inhibit tumor proliferation and prevent recurrence. In addition to the above, CSCs possess the capability of differentiation that enables them to give rise to the different cancer cells that are present in the tumor [27]. Furthermore, CSCs are also known to undergo transdifferentiation, that is, CSCs have the potential to transform into various types of multilineage cells and thereby regulate the growth of tumor [27]. Bussolati and colleagues found that renal CSCs were able to give rise to vascular endothelial cells (ECs) within the neoplastic mass when human renal CSCs were injected into an SCID mouse [28]. A similar differentiation of CSCs into endothelial cells and their angiogenic potential has been documented in various cancers, including liver cancer [29] and glioblastoma [30]. The differentiation capability of CSCs contributes to cellular variability within tumors, posing a significant challenge in treatment, as cells within the same tumor may respond differently to identical therapies. Understanding the mechanisms of CSC differentiation can help to prevent the formation of resistant cell lineages or enable the targeting of the most drug-resistant subpopulations for more effective treatment strategies. Perhaps the biggest challenge with regard to CSCs is that they are not easily killed by conventional chemotherapy treatments. Thus, knowing the mechanisms of CSCs’ chemoresistance is very important in the management and prevention of cancer relapse. CSCs employ several strategies for evading apoptosis, including increased DNA repair, upregulation of drug efflux pumps that can throw out chemotherapeutic drugs, and the ability to arrest in the G0 phase of the cell cycle, thus being less sensitive to therapies aimed at cycling cells [30,31]. These characteristics not only make it difficult to eradicate CSCs but also allow them to survive a round of chemotherapy and cause recurrence of the disease after apparent clinical remission [16]. These characteristics of CSCs stress the need to develop novel and specific approaches for the treatment of cancer that will be able to target these particular cells. More research must be conducted on the molecular mechanisms that are involved in the process of self-renewal, differentiation and resistance of CSCs in order to develop new therapies that will overcome the limitations of conventional cancer therapies.

CSCs exhibit unique characteristics, including self-renewal, differentiation, and resistance to conventional therapies, which drive tumor formation, progression, and relapse (Table 1). Their self-renewal ability, regulated by key pathways like Wnt, Notch, and Hedgehog, ensures that their population is maintained, while their differentiation into various cell types within the tumor contributes to cellular heterogeneity, complicating treatment. CSCs also demonstrate transdifferentiation, such as forming endothelial cells to support angiogenesis, further enhancing tumor growth. Resistance mechanisms, including DNA repair, drug efflux pumps, and cell cycle arrest, make CSCs resilient to standard chemotherapy, leading to recurrence. Understanding these mechanisms is crucial for developing targeted therapies that effectively eliminate CSCs and improve cancer treatment outcomes.

## 3. Molecular Markers and Signaling Pathways That Define CSCs Across Different Cancer Types

CSCs are a unique subpopulation of cells within tumors that exhibit self-renewal, differentiation, and tumorigenic potential. These characteristics allow CSCs to initiate, propagate, and sustain tumor growth, making them central to cancer progression, metastasis, and resistance to conventional therapies [32,33]. CSCs are distinguished from other cancer cells and normal stem cells by the expression of specific molecular markers and the dysregulation of key signaling pathways that are typically involved in maintaining stem cell properties. These molecular markers and signaling pathways not only help to identify CSCs but also play a significant role in the biological processes that drive their tumorigenic behavior.

A major feature of CSCs is their expression of surface markers, which vary depending on the type of cancer. In breast cancer, for instance, CSCs are commonly identified by the markers CD44+/CD24−/low and ALDH+ (aldehyde dehydrogenase), which are associated with their ability to initiate tumor formation and sustain tumor growth [34]. In melanoma, CD34+CD38− is a typical CSC marker, whereas in glioblastoma, the CD133 marker is predominantly expressed by stem/progenitor cells [35]. These markers not only assist in identifying CSCs but also serve as a reflection of the unique properties they exhibit, including their ability to self-renew, differentiate into diverse cell types, and contribute to tumor heterogeneity. For example, CSCs in various cancers have been shown to possess the ability to transdifferentiate into multiple cell types, including endothelial cells, thus supporting angiogenesis and enhancing tumor growth [36].

The self-renewal ability of CSCs is tightly regulated by several key signaling pathways that are essential for maintaining their stem-like characteristics. These pathways, which are often dysregulated in CSCs, include the Wnt/β-catenin, Notch, and Hedgehog pathways. The Wnt/β-catenin pathway plays a critical role in regulating cell proliferation and differentiation in both normal stem cells and CSCs [37]. In colorectal cancer, the Wnt/β-catenin pathway is often upregulated in CSCs, contributing to their self-renewal and expansion [38]. Similarly, the Notch signaling pathway, which is involved in cell fate decisions and stem cell maintenance, is aberrantly activated in CSCs from glioblastoma, breast cancer, and pancreatic cancer [39]. In these cancers, Notch signaling is linked to CSC self-renewal, survival, and resistance to differentiation, allowing CSCs to persist and contribute to tumor relapse. The Hedgehog pathway, another key regulator of stem cell property, has been shown to be involved in CSC maintenance in glioma, leukemia, and other cancers [26]. The aberrant activation of Hedgehog signaling promotes the expansion of CSCs and enhances their tumorigenic potential [26].

**Figure 2:**
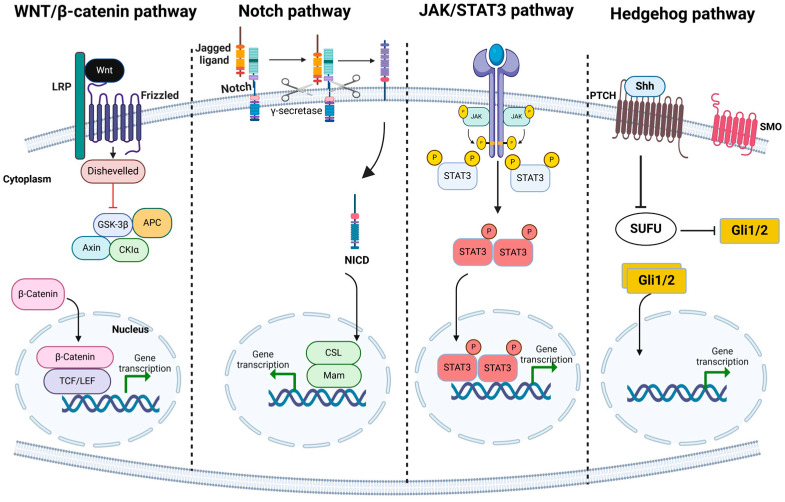
CSCs signaling pathways along with their signal transmission network. Several signaling pathways are pivotal in driving malignant transformation and tumor progression, particularly within the regulatory network of cancer stem cells (CSCs). The four major signaling pathways highlighted in this context are WNT/β-catenin, Notch, JAK/STAT3, and Hedgehog. The WNT/β-catenin pathway is regulated by the accumulation of inactive β-catenin, controlled by glycogen synthase kinase-3β (GSK-3β), with Axin and Dishevelled playing key roles. In the Notch pathway, the Notch intracellular domain (NICD) undergoes a series of three cleavage events, ultimately translocating into the nucleus to activate gene transcription. In the JAK/STAT3 pathway, signal propagation is mediated through downstream transphosphorylation events. Hedgehog signaling (Shh) is activated in CSCs through the inhibition of the SMO-dependent cleavage of Gli proteins, thereby promoting pathway activation.

The dysregulation of these signaling pathways in CSCs not only drives their stem-like properties but also contributes to the therapeutic resistance that makes CSCs particularly challenging targets for conventional cancer therapies. CSCs exhibit enhanced resistance to chemotherapy, radiotherapy, and targeted therapies through various mechanisms, including increased DNA repair, the upregulation of drug efflux pumps (such as ABC transporters), and the ability to enter a quiescent state in the G0 phase of the cell cycle. These mechanisms make CSCs less sensitive to treatments that target rapidly dividing cells, which are the hallmark of many conventional cancer therapies. Additionally, CSCs are capable of surviving rounds of chemotherapy and subsequently reinitiating tumor growth, leading to disease recurrence and metastasis. This resistance to conventional therapies underscores the importance of developing new treatment strategies that specifically target CSCs while sparing normal cells (Figure 2).

The heterogeneous nature of CSCs across different cancer types further complicates the development of effective, universal therapeutic strategies. While certain markers and pathways are commonly dysregulated across various cancers, the specific molecular features of CSCs can vary significantly depending on the tumor type. For instance, breast cancer CSCs may rely on different signaling pathways, compared to glioblastoma or pancreatic cancer CSCs, and the expression of surface markers may also differ between these cancers. This variability necessitates the development of personalized therapies that target the specific molecular characteristics of CSCs in each cancer type.

In addition to their role in therapy resistance, CSCs are implicated in the metastatic spread of tumors. By maintaining a population of self-renewing cells that can differentiate into various cell types, CSCs contribute to the cellular heterogeneity of the tumor, which enables the tumor to adapt and metastasize to distant organs. The ability of CSCs to form new tumors when transplanted into immunocompromised mice further emphasizes their role in tumor initiation and progression. These findings underscore the importance of targeting CSCs in the development of effective cancer therapies that not only target the bulk of the tumor but also eradicate the root cause of tumor growth and recurrence.

Overall, the molecular markers and signaling pathways that define CSCs play a pivotal role in their ability to drive tumorigenesis, metastasis, and resistance to therapy. Understanding the specific markers and pathways associated with CSCs in each cancer type is essential for the development of targeted therapies that can effectively eliminate CSCs and prevent cancer relapse. While significant progress has been made in identifying CSC-specific markers and signaling pathways, further research is needed to fully understand the complex molecular mechanisms underlying CSC biology. This knowledge will be crucial for developing novel therapeutic strategies that can target CSCs more effectively and improve cancer treatment outcomes. Additionally, a deeper understanding of the heterogeneity of CSCs across different cancers will be essential for the development of personalized treatment approaches that can better address the challenges posed by CSCs in cancer therapy.

CSCs are characterized by specific molecular markers and signaling pathways that distinguish them from other tumor and normal stem cells. These markers, such as CD44+/CD24−/low and ALDH+ in breast cancer or CD133 in glioblastoma, are critical for CSC identification and tumor initiation. Key signaling pathways like Wnt/β-catenin, Notch, and Hedgehog regulate CSC self-renewal and differentiation, often becoming dysregulated in cancers such as glioblastoma, colorectal, and breast cancer. Targeting these markers and pathways offers promising strategies to inhibit CSC-driven tumor growth, recurrence, and metastasis (Table 2).

## 4. Techniques for Isolating and Studying CSCs in the Laboratory and Clinical Settings

It has been observed that CSCs are present in small numbers in tumor tissues and account for 0.01% to 2% of the total tumor cells [32]. Also, CSCs and normal stem cells have been found to express similar transcription factors and signaling pathways [40]. Therefore, developing CSCs from tumor samples and identifying the same is a very complicated process. CSCs can be investigated by various techniques and each of them offers unique information regarding the biology of CSCs and their role in cancer. CSCs have been identified in different cancer cells with different biomarkers. Flow cytometry and cell sorting are the frequently used methods which employ fluororeacted antibodies to bind to certain CSC surface markers [41]. This enables the precise identification and separation of CSCs from the main tumor population, making it possible to undertake further studies on them. Sphere formation assays use the ability of CSCs to grow in non-adherent and serum-free conditions, which results in the formation of spheroid. This process measures both the ability of the CSCs to undergo renewal and to enrich cultures of CSCs for further analysis [42].

**Figure 3:**
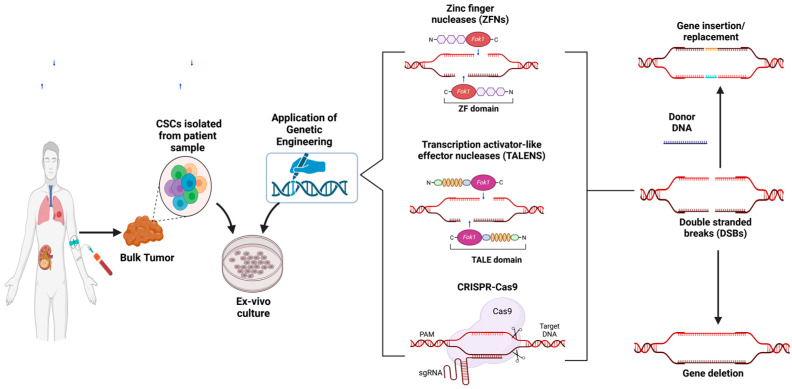
Schematic representation of application of gene-editing tools in CSC research. The initial step involves isolating CSCs from a patient’s tumor sample, followed by their expansion in an ex vivo culture system. Genetic engineering tools, such as Zinc Finger Nucleases (ZFNs) and Transcription Activator-Like Effector Nucleases (TALENs), are employed to target specific genomic sites using their respective DNA-binding domains—zinc fingers and transcription activator-like repeats. The CRISPR-Cas9 system achieves site-specific targeting through the recognition of a protospacer adjacent motif (PAM), highlighted in red, and the complementary sequence of the single guide RNA (sgRNA), shown in blue. Once the target site is identified, the catalytic subunits, either the Flavobacterium okeanokoites type IIS restriction enzyme (FokI) or CRISPR-associated protein 9 (Cas9), induce a double-strand break (DSB). This break can be repaired via non-homologous end joining (NHEJ), which may result in insertions or deletions, or through homology-directed repair (HDR) using a DNA template, enabling the introduction of precise genetic modifications. The upward arrow represents gene insertion or replacement, where donor DNA is integrated into the genome at the DSB site. The downward arrow indicates gene deletion, where DSBs are repaired without donor DNA, resulting in the removal of genetic material.

Taylor et al. isolated CSCs from different kinds of neurological tumors in this colony formation assay [43]. More knowledge is acquired through immunohistochemistry (IHC) and immunofluorescence (IF) assays, which involve the labeling of tissues or cells with antibodies to CSC markers [44,45]. This proves the presence of CSCs in the tumor microenvironment while giving information of where exactly they are located and in what numbers. The transfer of CSCs into immunocompromised mice is regarded as the classical approach to determine CSCs’ tumorigenic potential due to their ability to generate tumors [46]. RNA sequencing and proteomics analyses offer the understanding of the variability in gene and protein expression profiles of CSCs, which define their peculiar characteristics [47,48]. Genetic manipulation methods, like CRISPR-CAS9 and RNA interference (RNAi), help in controlling gene expression in CSCs, which finally helps in identifying the essential genes and pathways required for CSC function and proliferation [49,50,51]. Thus, drug screening tests are very vital in the identification of drugs that can affect CSCs and the effect that these drugs will have on tumor cell survival, growth, and differentiation. These techniques provide a basis for the development of novel therapies against CSCs, thus preventing cancer relapse and progression (Figure 3). However, the continuous improvement and optimization of these approaches are necessary to recognize the details of CSCs and their role in cancer.

Isolating and studying CSCs is a complex process due to their scarcity and similarities to normal stem cells. Advanced techniques like flow cytometry, cell sorting, and sphere formation assays enable the precise identification and enrichment of CSCs (Table 3). Immunohistochemistry and immunofluorescence offer insights into CSCs’ location and distribution within the tumor microenvironment, while RNA sequencing and proteomics reveal their unique gene and protein profiles. Functional studies using genetic tools like CRISPR-Cas9 and RNA interference highlight essential pathways driving CSC proliferation. These methods are pivotal for drug screening and the development of novel therapies targeting CSCs, offering hope to prevent tumor relapse and progression.

## 5. CSCs and Tumor Heterogeneity

Tumor heterogeneity refers to the diversity of cancer cells within a single tumor and between different tumors of the same type. This heterogeneity is largely driven by CSCs, which contribute to both inter-tumoral and intra-tumoral diversity. CSCs possess pluripotency and self-renewal capabilities, which allow them to differentiate into various types of cancer cells with distinct characteristics [63]. These cells vary in terms of genetic makeup, phenotypic features, and functional properties, contributing to the tumor’s complex biology. The ability of CSCs to generate diverse cell populations enables tumors to adapt dynamically to their environment, particularly in response to selective pressures such as therapy, leading to a constantly changing tumor composition. TME further influences the heterogeneity by shaping the behavior of CSCs and their ability to interact with other tumor cells and stroma.

The presence of heterogeneous populations of cancer cells within the tumor makes it difficult for conventional therapies to eradicate the entire tumor. Not all cancer cells respond uniformly to treatment, and this variability is a major challenge in effective cancer treatment. Therefore, understanding tumor heterogeneity is critical in developing strategies to target both the bulk of the tumor and the CSCs that sustain its growth and survival.

### 5.1. Role of Tumor Heterogeneity in Treatment Resistance

- Mechanisms of treatment resistance: CSCs play a significant role in promoting tumor treatment resistance. This resistance is attributed to several features inherent to CSCs, including the following:
- Quiescence: Many CSCs exist in a dormant or slow-dividing state, making them less susceptible to treatments like chemotherapy or radiation, which primarily target rapidly dividing cells.
- Enhanced DNA repair mechanisms: CSCs have efficient DNA repair pathways that enable them to survive the DNA damage caused by therapies.
- Drug efflux pumps: CSCs often express high levels of drug efflux pumps, such as P-glycoprotein, which actively pump out therapeutic agents, reducing their effectiveness.

Due to these characteristics, CSCs often survive initial treatment regimens that target proliferating tumor cells. After the bulk of the tumor is reduced, the surviving CSCs can reinitiate tumor growth, leading to recurrence. Furthermore, CSCs have the potential to acquire additional genetic mutations or adapt to therapy-induced stress, making them more resistant to subsequent treatments.

Tumor heterogeneity also plays a significant role in the evolution of drug-resistant clones. As treatment pressure is applied, tumors may evolve by selecting for clones that are naturally resistant to the therapy. These resistant clones may possess specific genetic mutations or adaptive changes that enable them to survive and continue to proliferate despite treatment. This process not only hampers the initial therapy but also complicates subsequent treatments, as the tumor evolves to form new resistant subpopulations.

### 5.2. Role of Tumor Heterogeneity in Disease Progression

Tumor heterogeneity also contributes to cancer progression through its impact on metastatic potential [64]. Different subpopulations of CSCs exhibit varying abilities to invade surrounding tissues and migrate to distant organs, where they can establish secondary tumors. These metastatic cells may retain some features of the primary tumor while adapting to the new microenvironment, further complicating treatment. The metastasis process is facilitated by the diverse functional capabilities of CSCs, which allow for the formation of metastatic niches and the ability to survive and thrive in distant organs.

As the tumor evolves and spreads, the microenvironment of metastatic lesions may differ from the primary tumor, requiring different treatment strategies. This makes metastasis an additional challenge in cancer therapy, as the heterogeneous nature of both primary and metastatic tumors demands a more personalized approach to treatment.

### 5.3. Challenges Posed by Tumor Heterogeneity in Identifying Molecular Profiles

The heterogeneity of tumors also complicates the identification of accurate molecular profiles that are necessary for guiding targeted therapies. A biopsy sample may not always represent the full genetic composition of the tumor, leading to potential misinterpretations of the tumor’s molecular characteristics. This limitation is particularly true for tumors with extensive intra-tumoral heterogeneity, where the genetic makeup of different tumor subpopulations may vary significantly. Inaccurate molecular profiling due to tumor heterogeneity may result in the selection of less effective treatments, as targeted therapy may not address the full spectrum of tumor cell populations present within the tumor. This highlights the need for more comprehensive and dynamic diagnostic techniques that can capture the full diversity of tumor cells.

### 5.4. Strategies to Overcome Tumor Heterogeneity and Enhance Treatment Efficacy

To address the challenges posed by tumor heterogeneity, several strategies are being explored:

- Targeting CSC populations: Directly targeting CSCs and their niche within the tumor could help eliminate the cells responsible for tumor initiation and relapse.
- Combination therapies: Multi-drug approaches that target different subpopulations of cancer cells within the tumor could reduce the likelihood of resistance and improve overall treatment efficacy.
- Adaptive treatment strategies: Treatment plans that can be adjusted dynamically in response to the changing tumor landscape may help counter the evolving nature of tumor heterogeneity.

Oct-4 (Octamer-binding transcription factor 4) and Nanog are pivotal transcription factors that are crucial for sustaining the self-renewal, pluripotency, and undifferentiated state of CSCs, thereby contributing significantly to tumor heterogeneity, progression, and therapeutic resistance [65]. Oct-4, a member of the POU family of transcription factors, regulates key genes involved in cell cycle regulation, survival, and proliferation. It plays a vital role in maintaining CSCs by interacting with major signaling pathways, such as Wnt, Notch, and Hedgehog, which are essential for stem cell properties. The increased expression of Oct-4 is strongly correlated with aggressive tumor behavior, including enhanced metastatic potential, therapy resistance, and poor prognosis across various cancers, including breast, ovarian, prostate, and lung cancers. Nanog, a homeobox transcription factor, works in concert with Oct-4 to reinforce the stem-like characteristics of CSCs [66]. It modulates pathways such as STAT3, AKT, PI3K, and TGF-β, thereby promoting tumor progression, survival under harsh conditions like hypoxia, and resistance to immune surveillance. Elevated Nanog expression is seen in a wide range of cancers, including glioblastoma, hepatocellular carcinoma, and colorectal cancer, and is associated with increased metastatic spread, relapse, and chemoresistance [67]. Both Oct-4 and Nanog are not only critical markers for identifying CSC populations but also key drivers of tumor plasticity, enabling CSCs to adapt to changing environments and therapeutic challenges [67]. Their functional redundancy and complex interactions with other stemness-related factors highlight their importance in CSC biology and their potential as therapeutic targets to overcome CSC-driven tumor heterogeneity and resistance, offering promising avenues for more targeted and effective cancer therapies.

**Figure 4:**
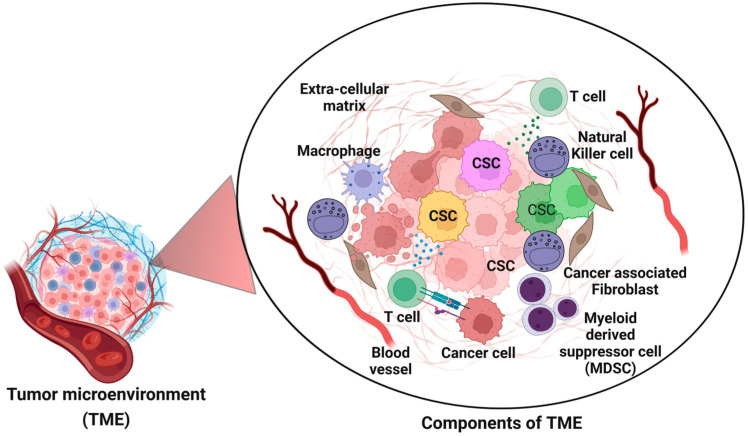
Intra-tumoral heterogeneity, development of resistant tumors and therapeutic potential of anti-CSC drugs. Schematic representation of the diverse subpopulations of cancer within tumors, contributing to tumor heterogeneity. The figure illustrates how CSCs drive the development of drug-resistant and metastatic tumors, highlighting their role in tumor progression and therapy failure. A potential therapeutic strategy targeting CSC-specific pathways or markers is depicted, aiming to eradicate CSCs while sparing normal cells. This approach emphasizes the potential of CSC-specific drugs alone and/or in combination with conventional anti-cancer therapy, to prevent tumor recurrence and metastasis by effectively targeting the root cause of malignancy.

CSCs play a pivotal role in tumor heterogeneity, driving diversity in genetic, phenotypic, and functional traits within and across tumors (Figure 4). This heterogeneity enables CSCs to evade conventional therapies, promoting treatment resistance, disease progression, and tumor recurrence. CSCs adapt dynamically to therapy-induced pressures, generating resistant clones that complicate treatment and metastasis. Accurate molecular profiling and adaptive therapeutic strategies targeting CSCs and their mechanisms are essential to counter heterogeneity and improve cancer outcomes. By focusing on CSC-driven processes, novel approaches can minimize disease progression and enhance the efficacy of cancer therapy.

## 6. The Dynamic Interplay Between CSCs and the Tumor Microenvironment

The communication between CSCs and the TME plays a critical role in the growth, progression, spread and treatment resistance of cancer [68]. The TME comprises many cell types, such as immune cells fibroblast, endothelial cells and the extracellular matrix, which provide a suitable milieu for CSCs [69]. CSCs communicate with the TME through a plethora of signaling pathways, cytokines, and growth factors that enable the CSCs to regulate their own proliferation, differentiation, and death [70]. CSCs secrete various factors that recruit and modulate stromal cells, including fibroblasts and immune cells, to promote the development of a tumor-supportive microenvironment [71]. The reprogrammed stromal cells secret some growth factors and cytokines that support CSCs and also protect them from the conventional therapeutic drugs.

The hypoxic conditions that are often found in tumors due to poor vascularization also play a very important role in this relationship [72]. In CSCs, hypoxia-inducible factors (HIFs) are overexpressed in response to low oxygen levels, which leads to the enhancement of stemness characteristics, angiogenesis, and metabolic adaptation [73]. Hypoxia also regulates CSCs signaling pathways (Wnt and Notch) which induce the EMT, thereby promoting the stemness of CSCs and increasing their invasiveness and resistance to radiotherapy and chemotherapy [74]. This interaction increases CSC survival and tumor progression [75]. The TME can also help CSCs to escape from immune system detection [76]. These cells control the immunological environment by secreting cytokines that suppress immunity or by expressing co-inhibitory signals that prevent the host from eliciting an adequate immune response against the tumor. CSCs have to escape detection in order to survive and promote disease and this is where communication with the microenvironment comes in to play [77]. CSCs and their interaction with the TME are important in metastasis [78,79]. When CSCs are exposed to stimuli from the TME, they undergo EMT, which allows them to move and invade in a process that is necessary for metastasis [80]. When these cells arrive at a different part of the body, they interact with the new microenvironment to form metastatic niches, which results in the formation of more tumors. CSCs and the TME are interdependent and this relationship is critical in the development of tumors, making it crucial to develop therapies that target both the CSCs and their niche. The TME can also protect CSCs from conventional therapies, thus proving the need for new therapies that target both CSCs and their microenvironment. This interdependent relationship between CSCs and the TME is, therefore, important to understand so as to come up with better therapeutic strategies that can break this relationship and thus, prevent the growth of the tumor.

**Figure 5:**
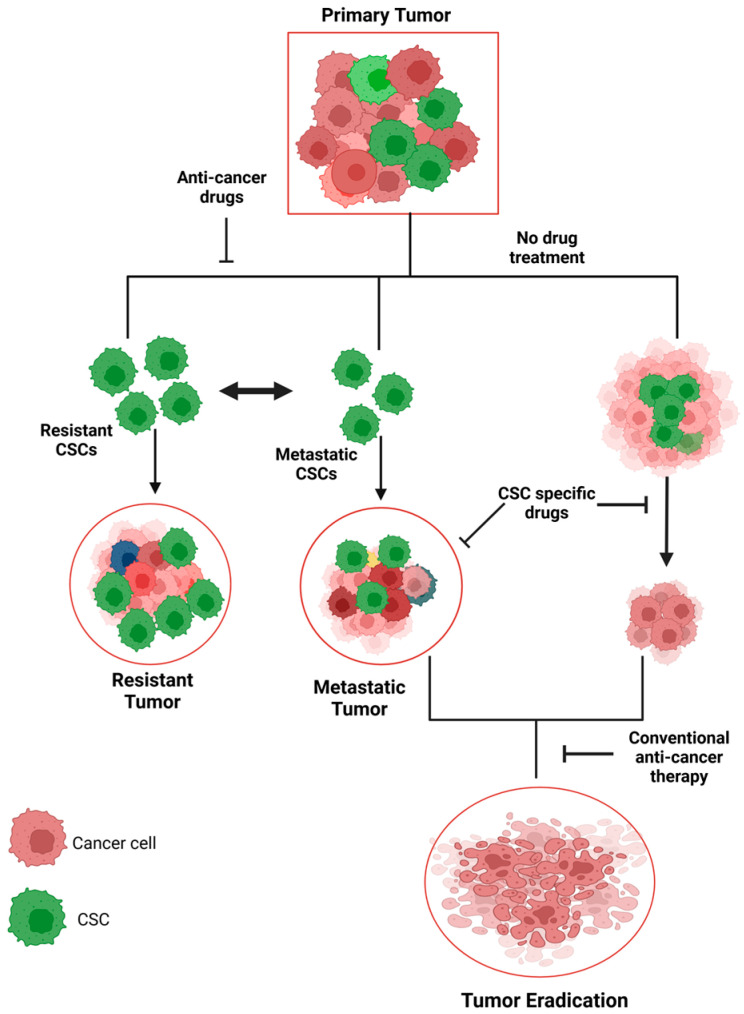
CSCs in tumor microenvironment. Schematic representation of the CSCs niche within the tumor microenvironment, highlighting the dynamic interactions between CSCs and surrounding stromal cells, immune cells, extracellular matrix components, and signaling molecules. These components, together, contribute to CSC renewal and maintain tumor malignancy.

The dynamic interplay between CSCs and the TME is crucial for cancer growth, progression, metastasis, and treatment resistance (Figure 5). The TME, comprising immune cells, fibroblasts, endothelial cells, and the extracellular matrix, provides a supportive niche for CSCs through signaling pathways, cytokines, and hypoxic conditions. This interaction enhances CSC stemness, promotes immune evasion, and facilitates EMT for metastasis. The TME also shields CSCs from conventional therapies, emphasizing the need for innovative treatments targeting both CSCs and their microenvironment. Disrupting this interdependent relationship holds promise for preventing tumor growth and improving therapeutic outcomes.

The following table provides a more comprehensive view, breaking down the complex interactions between CSCs and the TME, emphasizing their role in various aspects of tumor progression, metastasis, and resistance to treatments. The table also highlights the need for therapies targeting both CSCs and the TME to improve cancer treatment outcomes (Table 4).

## 7. CSCs in Metastasis and Recurrence

Such structures are essential in the encouragement of metastasis, which is one of the deadliest factors in cancers [14]. One crucial step in the metastatic process involves the migration of cancer cells from the primary tumor, and CSCs are thought to facilitate this migration in some way [92]. CSCs are involved in the progression of cancer in a sequential manner through a number of distinct phases which may be attributed to the properties of CSCs and their crosstalk with the TME. CSCs exhibit EMT, a process where the epithelial cells acquire some features of mesenchymal cells, allowing their overall cell migration and invasive capacities to be enhanced. This transformation is crucial for metastasis as it enables CSCs to detach from the main tumor, invade the surrounding tissues and enter into the bloodstream or lymphatic vessels [93,94]. EMT is mediated by many signaling pathways and microenvironmental factors, such as TGF-β, Wnt/β-catenin, and Notch, which are often dysregulated in CSCs [95,96]. Therefore, when circulating in the bloodstream, CSCs have high survival rates since they are able to withstand the shear stresses and evade immune system recognition [97]. These cell characteristics increase the cells’ chances of surviving in an environment as harsh the circulatory system. CSCs, when transported to a different part of the body, can go through a process called mesenchymal-to-epithelial transition where they are able to re-establish cell contacts and form micrometastases [91]. CSCs can remain latent for prolonged periods and this helps CSCs to avoid being detected and treated. This capability may lead to delayed recurrence when they resume function and start dividing SCDs in response to stimuli by suitable conditions in the surroundings. The retention of CSCs in a quiescent and extracellular state is regulated by several signals received from the intracellular TME that include the modulation of quiescence, angiogenesis, and immune surveillance [97]. The formation of secondary tumors is due to the re-emergence of CSC functions whereby these cells are able to proliferate and differentiate into different cell types, thereby forming a new tumor that often resembles the heterogeneity of the primary tumor [97]. CSCs communicate with the new stroma to regulate stromal and immune cells and thereby establish a microenvironment that is conducive for tumor growth and progression.

CSCs can also contribute to the formation of pre-metastatic niches in target organs even before the CSCs themselves arrive there [98]. These variables can therefore condition the remote tissue for tumor implantation by modulating the environment and promoting angiogenesis and suppressing immunological responses. For instance, exosomes released from CD105+ renal CSCs do enhance angiogenesis and aid in the progression of the kidney cancer to the lungs. This is carried out through endocytosis and the subsequent increasing of VEGF and MMP-2 in lung endothelial cells [99]. Also, the exosomes released from highly metastatic melanoma were found to enhance metastatic potential by stimulating the bone marrow hematopoietic progenitors to express the MET receptor tyrosine kinase. Thereafter, these progenitors were stimulated by HGF, leading to the conversion of BMDCs to progenitors that had enhanced migratory capacity and the potential of forming pre-metastatic niches [100]. Despite the increasing focus on the role of exosomes in CSCs and the TME in the progression of cancer, there is very little evidence regarding the role of CSC-derived exosomes in metastasis, which is worth further investigation.

CSCs play a pivotal role in metastasis and recurrence by driving cancer progression through EMT, enabling their detachment, invasion, and survival in the bloodstream. These cells can evade immune detection, endure harsh conditions, and establish micrometastases at distant sites via mesenchymal-to-epithelial transition. CSCs further contribute to recurrence through prolonged dormancy and reactivation under favorable conditions, forming tumors resembling the primary one. Additionally, CSC-derived exosomes condition remote tissues by promoting angiogenesis and immune suppression, facilitating pre-metastatic niche formation. Understanding these mechanisms highlights the need for targeted therapies to disrupt CSC-driven metastasis and recurrence (Table 5).

## 8. Role of CSCs in Tumor Relapse and Resistance to Therapy

CSC are very much involved in tumor relapse and therapy resistance, which is a long-term threat to the effectiveness of conventional cancer therapies. Tumor relapse is quite common and occurs when most of the tumor has been killed by the treatment but the CSCs are in a quiescent state [111]. There are several mechanisms that allow CSCs to survive. This enables them to evade therapeutic stress as a result of being able to go into a quiescent state. CSCs have better DNA repair capacity than normal cancer cells and, therefore, are able to overcome the damage that is caused by the treatments. They also have higher levels of drug efflux pumps through which the chemotherapeutic drugs are ejected from the cell, hence reducing the drug accumulation and potency [112]. There is a tendency that, after the treatment, the self-renewal capacity of cancer stem cells will lead to formation of a new tumor, thus causing a recurrence. The new tumor can regenerate and progress through the process of therapy due to genetic and epigenetic alterations that are brought on by the treatment. This force of evolution works in favor of the CSCs for the survival of the fittest, but the fittest cells are of the aggressive and resistant type, hence hindering the cure [113]. In addition, the ability of CSCs in regulating TME and angiogenesis also promote tumor recurrence [113]. The role of CSC in tumor relapse and therapy resistance highlights the need for new cancer treatments that are able to target these cells together with their microenvironment. Potential approaches may include the use of drugs that target CSCs’ particular pathways, treatments that make CSCs sensitive to conventional drugs, and immunotherapies that reverse the suppression by CSCs.

CSCs play a central role in tumor relapse and therapy resistance, posing significant challenges to conventional cancer treatments (Table 6). These cells evade therapeutic stress by entering a quiescent state, exhibiting superior DNA repair capabilities, and expelling chemotherapeutic drugs via efflux pumps. Following treatment, CSCs can self-renew, adapt genetically and epigenetically, and promote tumor recurrence through interactions with the TME and angiogenesis. Their survival mechanisms favor the emergence of aggressive, resistant tumors. Targeting CSC-specific pathways, sensitizing CSCs to conventional therapies, and employing immunotherapies are promising strategies to overcome these challenges.

## 9. Exploration of the Concept of “Dormant” CSCs and Their Impact on Disease Recurrence

Dormant CSCs are a type of CSCs which are in quiescent state with reduced mitotic and oxidative activities [117]. CSCs can also escape the side effects of conventional treatments since they can stay in a dormant state, thus not being affected by treatments that target rapidly dividing cells. Although the majority of the tumor may respond well to treatment and shrink or appear to be eliminated, latent CSCs can withstand these therapies [118]. Dormant CSCs are precursors that have a huge impact on disease relapse. Thereafter, once the therapeutic pressure is relieved, these dormant CSCs come out of dormancy and go into the active state. In reaction, they use the intrinsic capacity of self-renewal and differentiation to replace the tumor and lead to a relapse. This recurrence can be more invasive and less sensitive to the previous treatments because of the genetic and epigenetic alterations that may have occurred during the dormant period or in response to the treatment [119]. Furthermore, the ability of CSCs to maintain a dormant state within the TME, mediated by factors such as hypoxic conditions and the regulation of quiescence, significantly complicates the prediction of cellular interactions and the prevention of cancer recurrence [100]. Other signaling pathways that are also involved in the maintenance of the latent state include the stress response and survival pathways. The interval can range from several months to years, hence presenting a challenge in the management of the condition and underlining the importance of post-treatment follow up [120]. Hence, it is crucial to gain knowledge about the mechanisms that regulate the initiation and termination of dormancy for the development of therapeutic strategies that can lead to the eradication of dormant CSCs either by eradicating them at their latent state or by preventing their reactivation. Some strategies entail targeting the survival pathways that are only active in latent CSCs, altering the environment within which the CSCs reside in the tumor or through the use of immunotherapy to enhance the ability of the immune system in eradicating these cells [121]. The focus on quiescent CSCs shows that cancer biology is far from being understood and that new strategies and approaches in research and treatment are needed. To that end, understanding the mechanism of action of dormant CSCs and their role in disease relapse may enable the development of newer strategies for treatment, which can lead to permanent remission and improved outcomes for cancer patients.

Dormant CSCs remain in a quiescent state, evading conventional therapies targeting actively dividing cells and playing a pivotal role in disease relapse. These latent CSCs can survive treatment, reawaken when therapeutic pressure is relieved, and regenerate tumors with increased invasiveness and resistance due to genetic and epigenetic changes. Factors like hypoxia and TME interactions regulate their dormancy, making the prediction and prevention of relapse challenging. Understanding the mechanisms governing CSC dormancy and reactivation is critical for developing strategies to eradicate these cells, whether by targeting their unique survival pathways, altering their niche, or enhancing immune system responses. Advancing research in this area offers the potential for permanent remission and better cancer outcomes. The following table summarizes the research on dormant CSCs, their mechanisms of survival, and their role in cancer recurrence, offering insights into potential therapeutic strategies to target these elusive cells and prevent relapse (Table 7).

## 10. Strategies for Specifically Targeting CSCs, Including Novel Therapeutic Agents and Approaches

Due to the involvement of CSCs in tumor recurrence and chemoresistance, new strategies aimed at targeting these cells are of the utmost interest in cancer therapy. One of the strategies include designing treatments that target CSCs using surface markers that are unique for these cells. For instance, an in vivo study reported that the administration of a monoclonal antibody targeting CD44 resulted in a greater than 50% reduction in acute myeloid leukemia burden [127]. Importantly, CD44 is a regulator of several miRNAs that are involved in the regulation of CSCs. Another effective approach is to target key pathways which are essential for the function and growth of CSCs. Therapeutics that affect these pathways have produced promising outcomes in inhibiting the proliferation and survival of CSCs. In a study on the use of cyclopamine in blocking the Hh pathway in glioblastoma stem cells, Bar et al. noted that there was a reduction in the growth of adherent glioma cell lines with high Gli1 expression by 40–60% and no neurospheres were formed [128]. Differentiation therapy is a new approach which focuses on making CSCs differentiate into a smaller number of tumorigenic cells [129]. This method reduces the pool of CSCs and thus, makes the tumor more susceptible to conventional therapies, such as differentiation therapy. Immunotherapy is under consideration to target CSCs where vaccinations are performed to trigger the immune response against CSC markers or the CAR-T cell therapy [130]. Thus, the use of immunotherapy with immune-checkpoint inhibitors may help the body to break through the suppressive tumor environment that protects CSCs [131,132]. There is also another therapeutic approach that focuses on modulating the supportive niche of CSCs in the TME. This encompasses inhibiting angiogenesis which enables CSCs to obtain nutrients from the bloodstream, targeting stromal cells that produce growth factors required by CSCs or altering the extracellular matrix so as to prevent CSC mobility and distribution to other parts of the body [56,133]. At the moment, there are ongoing studies that are testing the effects of combination therapies in which CSC-targeted drugs are given together with conventional chemotherapeutic drugs or radiation therapy with the purpose of eradicating both CSCs and the bulk of tumor cells, with the intention of preventing the occurrence of tumor recurrence and progression [132,134,135].

Interestingly, the use of RNAi and CRISPR/Cas9 has been employed in the knock down or modification of genes that are essential to CSC and thus, make the CSC vulnerable to treatment [51,136]. Also, due to the current advancements in the field of nanotechnology, the delivery of drugs, genes, or RNA molecules to CSCs is being actively studied. Particles of the nanoparticle can be designed to target CSC-specific markers or to enhance the ability of the nanoparticles to penetrate the tumor microenvironment, thus enhancing the specificity and efficacy of CSC-targeted therapies [137,138]. For instance, Liu et al. recently developed a therapeutic liposomal nano formulation that was stealthy to target and eradicate CSCs along with non-CSCs [139]. In addition, the use of personalized therapy schedules which are tailored to the characteristics of CSCs in the tumor of a specific patient is crucial because CSCs may differ in various tumors and may change over time within the same tumor [140]. It is, therefore, important that there are continuous research and clinical trials in order to transform these novel pharmacological strategies for clinical practice.

### Challenges in Targeting CSCs Without Harming Normal Stem Cells

Eradicating CSCs while leaving normal stem cells unchanged is a challenging task because of the nature of these two cell types. Both CSCs and normal stem cells possess the property of self-renewal and differentiation, which are crucial for their role in cancer and for tissue regeneration, respectively [141]. Some of the therapies that have been designed to target CSCs may affect other normal stem cells as well, thus causing adverse effects and toxicity because of their similar characteristics. The major challenge that has been observed in the development of CSC-specific therapies is the fact that the two cell types are akin in their biology. For instance, CD44, CD133, and other markers, such as those mentioned earlier, are associated with CSCs, but these markers are also observed in normal stem cells in various organs, which makes it difficult to design targeted therapies that would not affect normal cells as well [142]. Therefore, it is important to identify markers or pathways that are differentially expressed or active in CSCs and not in conventional stem cells. Furthermore, a systematic knowledge of the differences in the expression levels of various cell types, their functional states, or signaling environments is also important. Interestingly, the other crucial characteristic of CSCs, which is their plastic and versatile nature, adds one more level of complexity. CSCs can alter their characteristics in response to stimuli or treatment, which may result in the alteration of the phenotype, the surface markers, and signaling pathways [143]. This plasticity allows them to become more like normal stem cells and thus, makes them harder to target specifically.

The microenvironment plays a critical role in maintaining CSC properties and can alter the behavior of stem cells in the same microenvironment [144]. Such therapies targeting the TME to target CSCs may also affect the microenvironment that supports normal stem cells, and hence, affect their function, and may cause tissue damage and stunted regeneration. To overcome these challenges, several strategies have been put in place. A strategy consists in identifying specific markers or weak points of CSCs, rather than those of normal stem cells, in order to design more specific therapeutics [145].

Secondly, targeting CSCs’ niche within the TME does not affect the niches of normal stem cells, possibly through local delivery approaches or using drugs with limited tissue diffusion [146]. Temporal targeting involves administering drugs at specific time points when the CSCs are in a unique state or easily distinguishable from the normal stem cells, which might reduce toxicity. Advances made in the knowledge of the differences between CSCs and normal stem cells, and the advancement made in the field of drug delivery systems and precision medicine, present a possibility of developing efficient CSC-targeted therapies with minimal effects on normal stem cells. Further research in this area is important in order to address these issues and to improve the safety and efficacy of cancer treatment.

Targeting CSCs presents a promising approach to overcoming tumor recurrence and chemoresistance. Innovative strategies include using monoclonal antibodies, pathway inhibitors, immunotherapies like CAR-T cells, differentiation therapies, and advanced drug delivery methods, such as nanoparticles tailored to CSC-specific markers. Cutting-edge technologies like CRISPR/Cas9 and RNAi are enabling precise gene modification to make CSCs more vulnerable to treatment. However, challenges remain in distinguishing CSCs from normal stem cells due to shared markers and plasticity, which complicates the development of specific therapies. Advances in understanding CSC biology, tumor microenvironment, and precision medicine hold potential for creating effective, safe, and personalized CSC-targeted treatments that minimize harm to normal tissues. The following table summarizes the various strategies for targeting CSCs, their outcomes, and the challenges faced in distinguishing CSCs from normal stem cells (Table 8). It highlights the potential of novel therapeutic approaches, including monoclonal antibodies, pathway inhibitors, immunotherapies, and advanced drug delivery technologies, while addressing the need for precision and minimal toxicity in treatment.

## 11. Review of Clinical Trials Focusing on CSC-Targeted Therapies and Their Outcomes

The current landscape of clinical trials of CSC-targeted therapies demonstrate the increasing focus on targeting the cause of tumor recurrence and the failure of conventional therapies [148]. These trials are conducted on a number of cancer types, such as breast cancer, colorectal cancer, lung cancer, brain cancer and others, and employ a number of approaches to target CSCs. The strategies entail the use of monoclonal antibodies, small molecule inhibitors, immunotherapies, and oncolytic viruses, all of which have been developed to interfere with the pathways that are important for CSC growth and maintenance [57]. Some of the features of these trials include the use of combination therapies, where the aim is to attack both the CSCs and the other tumor cells in order to avoid the development of escape mechanisms and enhance the outcome of the treatment.

However, there are also important barriers in the clinical trial landscape, such as the issue of CSC markers to determine the eligibility of the patients, the variability of CSCs in different cancers and the CSCs, and thus, the requirement to identify the right balance between the effectiveness and the side effects. Thus, some of the trials have shown positive outcomes where there is a reduction in tumor size and the rates of relapse while others are still in the early stages of the trial to determine the safety of the drug and the right dosage to be administered. Altogether, the current clinical trials are considered as important progress toward the incorporation of CSC-based therapies into conventional cancer treatment, improving the possibility of current treatment strategies and giving a light of hope to cancer patients. The details of the clinical trials that focus on CSC-targeted therapeutics, their outcomes, limitations and the practical implications are presented in Table 9.

The landscape of clinical trials targeting CSCs reflects growing efforts to address tumor recurrence and the limitations of conventional therapies. These trials span various cancer types, employing innovative approaches like monoclonal antibodies, small molecule inhibitors, immunotherapies, and oncolytic viruses to disrupt CSC-specific pathways. Combination therapies are a key focus, aiming to target both CSCs and bulk tumor cells to prevent escape mechanisms and improve treatment outcomes. While some trials report promising reductions in tumor size and relapse rates, challenges like patient eligibility, CSC variability, and balancing efficacy with side effects remain significant. Despite these hurdles, CSC-targeted therapies represent a hopeful step toward integrating advanced treatments into standard cancer care.

## 12. Overcoming Therapeutic Resistance Mediated by CSCs

### 12.1. Mechanisms of Resistance in CSCs to Conventional Chemotherapy and Radiation Therapy

Due to the presence of numerous mechanisms, CSCs are not sensitive to conventional chemotherapy and radiation therapy [157]. They can therefore move into a quiescent state where they are less likely to be affected by treatments that are designed to target rapidly dividing cells. Additionally, CSCs possess an enhanced DNA repair mechanism, enabling them to efficiently repair damage induced by various therapeutic interventions [158]. They also contain more drug efflux pumps that eliminate chemotherapeutic agents from the cells, thus reducing the efficacy of the drugs [12]. These mechanisms enable the persistence of CSCs during the course of treatment, which may lead to the recurrence and progression of the tumor. CSCs also resist conventional therapies through the expression of several key signaling pathways, including Wnt/β-catenin, Notch and NF-κB [159]. These pathways are involved in therapy resistance since they regulate cell differentiation, survival, and stemness, thus making CSCs very difficult to treat with conventional chemotherapy and radiation therapy. Targeting these pathways is a possible way of overcoming the resistance that is brought about by CSCs, thus improving outcomes [160].

CSCs can also evade conventional treatment through the use of the Wnt/β-catenin signaling pathway, which is essential for the maintenance of their stem cell-like characteristics and survival (134). Wnt signaling pathway inhibition leads to the inhibition of the degradation of β-catenin, which then translocate to the nucleus to enhance the transcription of genes that promote cell growth and drug resistance [161]. This process shows that it is challenging to target CSCs as they possess the ability of resisting treatments that are effective for non-stem cell cancer cells. The CSCs’ dependence on the Notch pathway is imperative for the development of chemotherapeutic and radiation resistance [162]. Notch signaling has been found to enhance the properties of cancer stem cells, such as self-renewal and survival, enabling them to be more resistant to conventional cancer therapeutics. Such a pathway could be a good target to make CSCs more sensitive to therapy [163]. Additionally, the NF-κB pathway is also important in CSCs as it regulates inflammation and stem cell-related genes which enhance survival and drug resistance to chemotherapy and other treatments [164]. Interfering with NF-κB may interfere with these mechanisms, offering a possibility of a new therapeutic approach.

### 12.2. Approaches to Sensitize CSCs to Current Treatments and Prevent Resistance

The issue of therapeutic resistance associated with CSCs is one of the major concerns in cancer treatment. To make the current standard treatments for CSCs more effective, strategies aim at targeting the specific biological pathways and survival mechanisms of CSCs [165]. The Wnt/β-catenin, Hedgehog and Notch signaling pathways, which are essential for the self-renewal and proliferation of CSCs, may be blocked in order to make CSCs more sensitive to chemotherapy and radiation therapy [166]. Targeting the specific microenvironmental niches which are crucial for CSCs can alter the protective microenvironment and thus, make the CSCs more sensitive to treatment. It is therefore important to identify the molecular mechanisms that lead to CSCs’ resistance to enable the development of new therapeutic strategies. Genomic and proteomic technologies have developed over the years and can help in the identification of important resistance-related genes and proteins in CSCs [167,168]. With the identification of these mechanisms, it is possible to develop drugs that will counteract these resistance pathways and thus, make CSCs respond well to conventional therapies. Also, the use of nanotechnology to deliver drugs to CSCs only can minimize the damage to other cells and increase the efficacy of the treatment [169]. To overcome CSCs’ therapeutic resistance, it is important to implement a systematic approach that involves sophisticated scientific knowledge and innovative treatment strategies.

### 12.3. Potential of Combination Therapies in Effectively Targeting CSCs and Non-CSC Cancer Cells

The use of drugs to target both CSCs and non-CSC cancer cells has been found to be effective in the treatment of cancer therapeutic resistance. The use of many therapeutic drugs in combination to attack the different aspects of cancer cell biology results in a more comprehensive attack on the tumor, and this is achieved through combination therapies [132]. The probabilities of the tumor coming back or spreading can be reduced through the use of combination therapies that target both the CSCs, which are almost always resistant to conventional therapies, and the less specialized non-CSC tumor cells [132]. This method is very effective as it reflects the diversity of tumors, which may have several subgroups of cells that may respond differently to certain forms of treatment.

These combination therapies are designed to attack the specific vulnerabilities of CSCs while, at the same time, eradicating the majority of the tumor, which is composed of non-cancer stem cells [170]. Using chemotherapy together with drugs that interfere with important pathways of cancer stem cells, including Wnt, Notch, and Hedgehog, may lead to the enhanced killing of tumor cells [171]. Also, conventional treatments can be combined with targeted therapies, like tyrosine kinase inhibitors or monoclonal antibodies, to fight against both CSCs and non-CSCs [172,173]. This versatile strategy can affect the equilibrium between CSCs and non-CSCs in the tumor and, therefore, the probability of treatment resistance and tumor relapse will be low. Also, with the advancement in the field of precision medicine and identification of specific biomarkers for CSCs, there exists a possibility of combining therapy. Developing therapeutic plans according to the molecular profile of the tumor and characteristics of the CSC population ensures that the therapeutic strategies are more accurate and effective. Also, immunotherapies, such as checkpoint inhibitors or adoptive cell transfer therapies, can be combined with the targeted therapies against CSCs, which have great potential [174]. Immunotherapies can enhance the ability of the immune system to recognize and erase CSCs, making it a powerful adjunct to the targeted therapy [131]. Therefore, the sensible use of combination drugs is important in overcoming barriers posed by CSCs in terms of therapeutic resistance, with the hope of achieving better and longer lasting responses in cancer treatment.

CSCs contribute to therapeutic resistance through mechanisms like quiescence, enhanced DNA repair, and drug efflux pumps, which allow them to survive chemotherapy and radiation. Key signaling pathways, including Wnt/β-catenin, Notch, and NF-κB, play crucial roles in CSC survival and resistance, making them difficult to target with conventional therapies. Strategies to overcome this resistance include blocking these pathways, altering the CSC microenvironment, and using advanced technologies like nanotechnology and genomic profiling. Combination therapies, which target both CSCs and non-CSC tumor cells, show promise in reducing relapse and improving treatment outcomes, especially when integrated with immunotherapies and precision medicine.

## 13. Advances in Single-Cell Sequencing and Other Technologies for Dissecting CSC Heterogeneity

Due to the advancement in technology and the emergence of new techniques, the CSC research field is evolving rapidly with the help of a more accurate understanding of the characteristics of these cells. New tools like single-cell sequencing have helped the understanding of CSC heterogenicity [175]. This innovative method allows researchers to analyze the genetics and genomics of the individual CSCs in a tumor, which helps to identify the various clusters and their modes of operation [176]. When the analysis of such diversity at the individual cell level is conducted, it helps to identify specific molecular pathways that are important for CSCs’ viability and their ability to withstand therapy. Besides single-cell sequencing, there are other state-of-the-art techniques that are also important in CSC studies. Enhanced imaging techniques and in vivo lineage analyses enable the observation, as well as the tracking, of CSCs in their native tumor context, which provides helpful information regarding their behavior, their interactions with other cells and their response to treatments [177]. These technologies help in discharging the dynamic nature of CSCs and their flexibility towards various treatment challenges. Organoid culture systems and 3D bioprinting are gradually being utilized for the creation of more physiochemical cancer models, which allows for studies of CSCs in systems that are closer to in vivo conditions [178,179]. CSC biology and its significance in cancer therapy and progression, as well as drug resistance mechanisms, are now being explored with the help of improved technologies. Single-cell sequencing, in conjunction with spatial transcriptomics, enables the identification of CSCs within the tumor bulk and the mapping of cellular interactions between CSCs and their surrounding environment [180]. This method provides a better way of understanding the different types of CSCs and their potential to change, as well as the identification of new targets and biomarkers for monitoring and, consequently, for targeting CSCs. Since the use of these technologies is expected to improve and be made more easily accessible in the near future, there is a possibility that new vistas in the field of CSCs will open up and create new horizons in the management of cancer and support patients.

### 13.1. The Potential of CRISPR/Cas9 and Other Gene-Editing Tools in CSC Research

The advancement of CRISPR/Cas9 and other gene-editing tools has led to the beginning of a new chapter in CSC research, whereby there is a possibility of identifying and targeting these elusive cells in the most efficient manner [181]. CRISPR/Cas9 enables the targeted modifications of the genome, which assists researchers to determine the effects of alterations of specific genes and pathways that are involved in the maintenance, growth, and drug resistance of CSCs [51]. It is possible to understand the genetic mechanisms that underlie CSC biology and identify new targets for therapy by knocking down or, possibly, restoring the genes that are considered to be important for CSC viability [182]. The following is a precise edit that can be used to introduce reporter genes or fluorescent tags to CSCs to help in the tracking and assessment of cells within the complex tumor niche. This process provides useful data on their activities and interactions with other cells.

Besides CRISPR/Cas9, other gene-editing tools like TALENs and ZFNs can also be used to boost the options available for the study of CSCs [183]. These technologies can act as substitutes to CRISPR/Cas9 for genome editing and may have specific advantages in terms of specificity, efficiency, and applicability [184]. TALENs and ZFNs can be employed to study the role of genes in CSCs and to generate CSC models for drug screening, which can be used to understand CSCs’ heterogeneity [185]. Moreover, integrating gene editing with other next-generation analyses, including single-cell sequencing and elaborate imaging, increases the potential to profile CSCs at a high level of resolution with a focus on their genetic and epigenetic profiles, and their positioning and dynamics in space, in the tumor [186].

CSC research can be greatly benefited by gene-editing technologies and there is not only scope for their use in basic science but also in therapeutic applications as well. CRISPR/Cas9 can improve CSC-targeted therapeutics through knocking out genes that are related to drug resistance or by reversing the causative genes that drive tumorigenesis [187]. It also helps in enhancing the efficacy of immunotherapies by changing the immune cells to target and kill CSCs or by changing CSCs to express antigens that can be identified by the immune system [49]. It has been predicted that the advancements in gene-editing technology will advance the CSC field and create chances for novel strategies for cancer treatment and better outcomes for patients [188].

Despite their transformative potential, technologies like CRISPR/Cas9 and single-cell sequencing face several limitations and challenges in CSC research and therapeutic applications. CRISPR/Cas9, while a powerful tool for gene editing, can introduce off-target effects, leading to unintended genetic alterations that may complicate the interpretation of results. Additionally, the efficiency of delivery to CSCs remains a challenge, as these cells are often difficult to target with standard delivery systems. Furthermore, the possibility of compensatory gene activation or redundancy in signaling pathways may limit the effectiveness of CRISPR-based strategies. On the other hand, single-cell sequencing, while offering the unparalleled resolution of CSC heterogeneity, is often hindered by issues related to data complexity and high costs. The technical challenges of isolating pure CSC populations from heterogeneous tumor samples can lead to biased or incomplete genomic profiles. Additionally, the high dimensionality of data generated through single-cell sequencing can make it difficult to draw meaningful conclusions without sophisticated computational tools and bioinformatics expertise. Moreover, both technologies raise ethical concerns regarding their potential long-term impacts, particularly with germline editing in CRISPR/Cas9 and privacy issues in the large-scale data collection associated with single-cell sequencing. Overcoming these challenges requires refining these technologies, improving delivery methods, and enhancing data analysis approaches to fully harness their potential in CSC research and therapeutic development.

### 13.2. Role of Artificial Intelligence and Computational Models in Understanding CSC Behavior and Designing Therapies

Due to the fact that the behavior of CSCs is not easy to predict, the use of AI and computer models is increasingly used to determine the behavior of CSCs and to develop specific management plans [189]. The most recent advances include machine learning and deep learning algorithms that can analyze big data from the genomic, transcriptomic, and proteomic analyses of CSCs and identify patterns, biomarkers, and targets for therapy that may not be detectable by conventional approaches [190]. Advanced computational models, incorporating AI, machine learning, and deep learning algorithms, offer the capability to predict CSC responses to diverse therapeutic interventions, map the underlying mechanisms of resistance, and recommend optimized combination therapies to prevent or counteract resistance [191]. The use of AI can thus offer a systematic understanding of CSC behavior within the TME, including their interactions with other cells, hypoxia responses, and metabolic adaptations, which leads to the development of new ways of targeting these hard to catch cells.

Computational models are very vital in the development of therapies for drugs that target CSCs and also in the development of the best treatment plans that would minimize resistance [192]. It is possible for researchers to model the signaling pathways that define CSC properties and to predict the effects of targeting these pathways with potential therapeutics. In silico drug screening can greatly decrease the time and cost of the drug discovery process to identify potential leads that will require further testing [193]. There are AI-based patient stratification algorithms that can help to find individuals who will likely respond to CSC-targeted drugs and thus, boost the efficiency of cancer treatment. These technologies are expected to advance as they will help in the development of new and enhanced cancer treatments in CSCs and therapeutic strategies, thus improving patients’ outcomes.

Advancements in technologies like single-cell sequencing, CRISPR/Cas9 gene editing, and artificial intelligence (AI) are revolutionizing the study of cancer stem cells (CSCs). Single-cell sequencing allows for the in-depth analysis of CSC heterogeneity, identifying crucial molecular pathways for their survival and resistance to treatments. Gene-editing tools like CRISPR/Cas9 enable the targeted modification of genes involved in CSC maintenance, offering new therapeutic avenues. AI and computational models, leveraging big data from genomics and proteomics, help predict CSC behavior, resistance mechanisms, and optimize treatment strategies, paving the way for more effective cancer therapies and improved patient outcomes.

## 14. Ethical Challenges in CSC Research and Therapy Development

The ethical challenges in CSC research and therapy development are multifaceted, and they demand comprehensive consideration across scientific, medical, legal, and societal dimensions. These challenges are particularly pronounced due to the profound therapeutic potential of CSC-targeted therapies, which could revolutionize cancer treatment by addressing tumor recurrence, chemoresistance, and metastasis. However, the targeting of CSCs also introduces a range of ethical dilemmas, particularly related to off-target effects, genetic manipulation, accessibility, and equity of treatment. One of the primary concerns involves the off-target effects of CSC-targeting therapies. CSCs are considered to be responsible for tumor initiation and recurrence, yet they share several characteristics with normal stem cells, which are crucial for tissue regeneration. Normal stem cells, particularly those found in tissues like the bone marrow, skin, and intestines, are vital for maintaining cellular homeostasis and enabling tissue repair. CSC-targeted therapies, especially those involving the use of monoclonal antibodies or small molecules, often rely on surface markers that are also expressed in normal stem cells. This overlap creates a significant ethical concern that therapies designed to eliminate CSCs could inadvertently target and damage normal stem cells, leading to severe side effects, such as immune system suppression, impaired wound healing, or long-term tissue dysfunction. These potential risks highlight the need for developing highly specific therapies that can differentiate between CSCs and normal stem cells, ensuring that the latter are spared from unintended damage. Additionally, the rapid pace of genetic editing technologies, such as CRISPR/Cas9, used to study or modify CSCs raises profound ethical issues related to genetic manipulation. While CRISPR/Cas9 holds immense promise for enhancing the treatment of cancers by directly correcting mutations within CSCs, it also poses the risk of introducing unintended genetic changes that could have far-reaching consequences. The main ethical concern here is that genetic alterations made to CSCs or cancer-related genes could potentially affect the genome of normal cells as well, leading to harmful off-target mutations that may induce cancer or other diseases in healthy tissues. Moreover, there is the distinct possibility that such genetic modifications could have heritable effects if germline cells are involved, which would raise concerns regarding long-term effects on future generations. The ethical implications of germline editing, particularly the potential for unintended hereditary changes, have sparked heated debates globally, particularly in light of recent incidents where embryos were genetically modified for research purposes. These concerns underscore the need for stringent guidelines and rigorous oversight to ensure that genetic interventions do not inadvertently harm individuals or the society at large. Alongside these technical challenges, the accessibility and affordability of CSC-targeted therapies present another significant ethical dilemma.

With the growing trend of personalized medicine where treatments are tailored to the unique genetic makeup of a patient’s tumor, the cost of developing and administering such therapies is rising rapidly. While targeted treatments hold the promise of more effective and less toxic therapies, the high cost associated with these personalized treatments could exacerbate existing healthcare disparities. The affordability of cutting-edge cancer therapies often limits their availability to patients in low- and middle-income countries, where the access to the latest medical advancements is restricted by financial constraints, limited healthcare infrastructure, and a lack of skilled professionals. In high-income countries, the access to such treatments may also be influenced by factors such as insurance coverage and socioeconomic status. These barriers raise difficult ethical questions about justice and equity in healthcare, as the access to life-saving treatments should not be restricted based on one’s ability to pay or geographical location. Ensuring that CSC-targeted therapies are available to all patients, regardless of their financial status or where they live, requires a coordinated effort among researchers, healthcare providers, governments, and international organizations. The global healthcare community must collaborate to develop strategies that address these inequities, such as creating affordable drug pricing models, ensuring adequate healthcare resources, and promoting the equitable distribution of new therapies.

Regulatory frameworks and guidelines governing genetic research and therapies vary significantly across countries, reflecting differing cultural, legal, and societal attitudes toward issues such as genetic editing, stem cell research, and the ethical use of human biological materials. In countries like the United States, the regulatory landscape for genetic research and gene therapies is governed by institutions such as the National Institutes of Health (NIH) and the Food and Drug Administration (FDA). These organizations provide comprehensive guidelines for ethical genetic research, including requirements for informed consent, patient autonomy, and the ethical use of human stem cells and genetic material. However, the U.S. approach is sometimes critiqued for being heavily influenced by the private sector, with commercialization playing a significant role in shaping research priorities and therapeutic development. This reliance on private-sector funding and innovation can lead to discrepancies in the distribution of advanced therapies and may prioritize profit over patient well-being. In contrast, the European Union has one of the most stringent regulatory frameworks governing genetic research and gene therapies. The European Medicines Agency (EMA) oversees the approval and regulation of gene therapies, ensuring that products undergo rigorous preclinical and clinical testing to assess their safety and efficacy before they reach patients. The EU’s regulatory environment also places significant emphasis on ethical considerations, particularly human rights, patient consent, and transparency in the research process. However, the strictness of these regulations can sometimes hinder the rapid advancement of innovative therapies, as the approval process for new treatments can be slow and cumbersome. While the EU’s rigorous oversight is intended to ensure patient safety, critics argue that such regulations may impede the timely availability of potentially life-saving therapies. Meanwhile, countries like China and India have less stringent regulations in comparison, leading to concerns about the adequacy of ethical oversight in research and clinical applications. In China, the rapid pace of gene-editing research and stem cell-based therapies has positioned the country as a global leader in the field. However, this unregulated progress has raised ethical concerns about patient safety and the potential for exploitation, particularly with regard to the use of vulnerable populations in clinical trials. The absence of a robust ethical framework for governing genetic modifications, particularly in the context of CRISPR/Cas9 applications, raises significant concerns about the potential for abuse and harm. Similarly, in India, the regulatory framework for stem cell research and therapy development has been criticized for being underdeveloped and insufficiently enforced, leading to challenges in ensuring the ethical conduct of research. While India has made strides in advancing stem cell research, gaps in ethical oversight and clinical trial regulation have led to concerns about patient protection and the potential for unsafe or unproven therapies being introduced to the market. These disparities in regulatory frameworks underscore the need for international cooperation and the establishment of global ethical standards to govern genetic research, CSC-targeted therapies, and related technologies.

Establishing global guidelines and frameworks for the ethical conduct of research and development in CSC-targeted therapies can help ensure that advances in the field benefit all people, regardless of their geographic location or economic status. Such international collaboration could help mitigate the risks associated with the commercialization of therapies, minimize disparities in access to new treatments, and ensure that the long-term health consequences of genetic modifications are carefully considered. The ethics of CSC research and therapy development also requires an ongoing dialogue among researchers, ethicists, policymakers, and the public. As the field of CSC-targeted therapies continues to evolve, new ethical questions and challenges will inevitably arise, necessitating the continuous development and refinement of ethical guidelines. This dialogue is essential to ensure that the benefits of CSC-targeted therapies are maximized while minimizing potential harm and ensuring that these therapies are accessible, affordable, and equitable. In conclusion, ethical challenges in CSC research and therapy development encompass a broad range of issues, from off-target effects and genetic manipulation to the accessibility and affordability of personalized treatments. These challenges require careful consideration and the establishment of stringent ethical guidelines that are responsive to the needs of patients, researchers, and the broader global community. Ethical oversight must balance the promise of new therapies with the protection of patient rights and the prevention of harm. As such, an ongoing dialogue, collaboration, and the establishment of international standards will be critical in ensuring that the development of CSC-targeted therapies proceeds in a responsible and equitable manner.

Ethical challenges in CSC research and therapy development are significant and require careful consideration. The concerns include off-target effects and risks related to CSC targeting, as these cells share similarities with normal stem cells crucial for tissue regeneration. The use of advanced technologies like CRISPR/Cas9 raises issues of genetic editing and its potential long-term consequences. Additionally, the accessibility and affordability of personalized CSC-targeted therapies highlight the need for collaborative efforts to ensure that these advancements benefit society while maintaining ethical integrity. Ongoing discussions among ethicists, researchers, and policymakers are vital to address these challenges.

## 15. The Future Landscape of CSC-Targeted Therapies and Personalized Medicine

It can be stated that the future of CSC-targeted therapeutics is on the cusp of a major advancement where the concept of personalized medicine rules the market and holds the key to evolving cancer treatment strategies. As the available information regarding CSCs and their role in cancer progression, relapse, and conventional therapy failure increases, the potential of developing novel drugs that target these cells also increases [140]. The shift towards therapeutics targeting CSCs is expected to bring about a new era of cancer therapy. These therapies are planned to not only remove the main tumor cells but also eliminate CSCs, which are often the main cause of cancer relapse. Technological advancements in genomes, proteomics, and bioinformatics enable a detailed study of CSCs in individual tumors and thus, the development of tailored therapies that target the specific vulnerabilities of CSCs in the tumor of each patient [194,195].

CSC-targeted drugs, within the context of personalized medicine, employs elaborate diagnostic procedures to determine the specific markers, as well as signaling pathways, that nourish CSCs in different types of cancer [196]. This precise method allows for the identification of therapeutic drugs that are more likely to work on the patient’s specific CSCs, thus increasing the chances of the therapy working while, at the same time, minimizing the side effects. CSC-specific biomarkers will play a crucial role in clinical practice, enabling the assessment of therapy efficacy and early detection of resistance. It is anticipated that combination therapies targeting various aspects of CSC biology, such as self-renewal, differentiation potency, and niche interactions, will become integral to future cancer management strategies. These approaches aim to address CSC-driven resistance comprehensively, offering a more effective framework for combating cancer [197]. This relapse strategy could be instrumental in enhancing future cancer management by integrating personalized medicine (PM) approaches with CSC-targeted therapies. The use of artificial intelligence (AI) and machine learning (ML) algorithms is expected to significantly reduce the chances of relapse. By analyzing large datasets from genomic, transcriptomic, and proteomic analyses of CSCs, AI and ML can identify patterns and biomarkers that may not be easily detected by human researchers. This approach will improve the accuracy of CSC identification and therapeutic intervention, helping to overcome challenges in cancer treatment [198,199]. This may lead to the development of more complex models that can be used to estimate the likelihood of certain patients’ response to CSC-targeted therapies so that the treatment plans can be tailored to the patients. Substantial advancements are anticipated to enhance the precision of these therapies, facilitating the identification of novel therapeutic targets in CSCs. Moreover, these developments are expected to improve the incorporation of tumor microenvironmental factors into the design of more effective and targeted treatment strategies. CSC-targeted drugs in the field of personalized medicine have the potential of enhancing patients’ outcomes in cancer.

The future of CSC-targeted therapies lies in the shift toward personalized medicine, with novel drugs aiming not only to remove tumor cells but also to eliminate CSCs, the primary cause of cancer relapse. Advancements in genomics, proteomics, and bioinformatics enable tailored therapies that target specific vulnerabilities of CSCs, improving treatment effectiveness and minimizing side effects. Personalized medicine, combined with AI and machine learning, will enhance CSC identification and therapeutic precision, reducing the risk of relapse and improving patient outcomes. The integration of CSC-specific biomarkers and combination therapies targeting CSC biology is set to revolutionize cancer management and offer more effective treatment strategies.

## 16. Key Research Areas and Collaborations Needed to Advance the Understanding and Treatment of CSCs

To better understand and deal with CSCs, it is important that there is a clear strategy that is developed with input from various fields of research and different scientific disciplines. There is one important element, and that is to understand the molecular and genetic mechanism of CSCs, where the help of molecular biologists, geneticists and bioinformaticians is required. This includes identifying the pathways that are involved in the regulation of CSC proliferation and the identification of the mechanisms that are behind the resistance of CSCs to conventional therapies, as well as the identification of CSC markers on the surface and their metabolic profile [200]. It is also important to understand the role of the TME in supporting CSC survival and helping CSCs to escape from immune system detection [201]. This calls for the collaboration with immunologists, pathologists and experts in tumor biology to develop strategies that will be capable of uncovering the relationship between CSCs and their niche.

A comprehensive understanding is crucial for developing targeted therapies that selectively eliminate CSCs without adversely affecting normal stem cells. To advance CSC-targeted therapeutics, it is imperative to integrate state-of-the-art technologies and novel therapeutic approaches, which involves close cooperation between nanotechnologists, drug developers, and clinical oncologists [202]. The use of advanced drug delivery systems, for instance, nanoparticles, to home in on CSCs, along with the use of gene-editing tools such as CRISPR/Cas9 for the precise modulation of CSCs, demonstrate the cross-disciplinary work that is needed [203]. Furthermore, the possibility of applying immunotherapy for targeting CSCs proves the importance of the cooperation between cancer immunologists and clinical researchers in the development of novel CSC-targeted immunotherapeutic approaches [204]. Therefore, there is a need to establish a strong collaboration between academic institutions, research facilities and the pharmaceutical industry in order to translate basic research findings into therapeutic interventions. Thus, through multidisciplinary collaborative research, the field can continue to overcome the challenges posed by CSCs and develop better and more lasting cancer treatments.

Advancing the understanding and treatment of CSCs requires a collaborative approach across multiple scientific disciplines. The key areas of focus include uncovering the molecular mechanisms driving CSC proliferation, resistance to therapies, and survival in the tumor microenvironment. Collaborative efforts between molecular biologists, geneticists, immunologists, oncologists, and nanotechnologists are essential for developing targeted therapeutics, including gene-editing tools and advanced drug delivery systems. Establishing strong partnerships between academic institutions, research facilities, and the pharmaceutical industry will enable the translation of basic research into effective cancer treatments, improving outcomes for patients.

## 17. Polar View

Several critical issues that are crucial for the effective implementation of strategies aimed at identifying and overcoming the limitations of CSCs have been identified. The variability and plasticity of CSCs remain key obstacles, potentially reducing the efficacy of interventions targeting specific markers or pathways. While therapies that utilize genetics and nanotechnology offer a high degree of precision, they also raise safety concerns.

The development of any new therapeutic strategies from the experimental level to clinically useful treatments involves a prolonged process and numerous tests to establish both effectiveness and safety. Moreover, the creation of novel and personalized therapies could result in treatments that are not only costly but also potentially inaccessible to many, thus raising ethical questions regarding the distribution of advanced cancer treatments.

Additionally, the requirement for combinatorial approaches to target both CSCs and the main tumor cell population complicates treatment schedules, potentially making administration more challenging and increasing the risk of severe side effects. This means that the benefits of targeting CSCs must be weighed against the practical challenges that would be encountered in a clinical setting.

The development of CSC-targeted therapies represents a promising area of research in oncology, offering the prospect of developing more effective and less toxic cancer therapies. Future studies and advancements in this field are crucial to address the current issues and achieve the desired outcomes. These approaches are designed to specifically target the cells believed to be responsible for tumor progression and recurrence, thereby potentially limiting the impact on non-cancerous cells and reducing the incidence of side effects, a significant improvement over conventional chemotherapy.

Concepts such as immunotherapy and the tumor microenvironment emphasize viewing cancer not merely as a mass of malignant cells but as a complex system. The use of cutting-edge technologies like CRISPR/Cas9 and nanotechnology highlights significant advancements in cancer therapy research, aimed at improving the precision and efficacy of CSC-targeted treatments.

However, CSCs are known to vary in their characteristics within and across different cancer types, making it challenging to identify a universal therapeutic target. This variability suggests that therapies may need to be highly individualized, which could be costly and less accessible. The safety and specificity of interventions, particularly those involving genetic modifications and nanotechnology, raise concerns about potential side effects and long-term impacts, which must be thoroughly evaluated in clinical trials.

Moreover, the transition from laboratory research to clinically applicable treatments involves numerous challenges, one of the most significant being the demonstration of the efficacy and safety of these strategies. The complexity of multiple targeted therapies and their respective mechanisms of action and side effects can lead to intricate treatment schedules and complex patient care scenarios. Despite these challenges, current efforts to target CSCs are recognized as one of the most crucial directions in the fight against cancer. They promise to develop treatments that are less likely to result in cancer recurrence and have fewer side effects, thus enhancing patient outcomes.

The development of CSC-targeted therapies holds great promise but faces significant challenges, including the variability and plasticity of CSCs, which can limit treatment efficacy. While advanced technologies like CRISPR/Cas9 and nanotechnology offer precise targeting, safety concerns and the complexity of combinatorial approaches complicate clinical implementation. Personalized therapies may be costly and less accessible, raising ethical questions about their distribution. However, despite these challenges, CSC-targeted strategies are crucial in the fight against cancer, potentially offering more effective and less toxic treatments with fewer side effects and improved patient outcomes.

## 18. Conclusions

CSCs are central to understanding tumor progression, therapeutic resistance, and disease recurrence, making them a crucial focus of contemporary oncology research. This review has comprehensively explored the multifaceted nature of CSCs, beginning with their defining characteristics of self-renewal, differentiation, and capacity to sustain tumor heterogeneity. By elucidating the molecular markers and signaling pathways, such as Wnt, Notch, and Hedgehog, this manuscript highlights the molecular underpinnings that define CSCs across diverse cancer types, offering critical insights into their biological complexity. Techniques for isolating and studying CSCs, both in laboratory and clinical settings, have further emphasized the need for precision in identifying and targeting these elusive cells.

The interplay between CSCs and the TME emerges as a dynamic determinant of tumor behavior, fostering metastasis, therapeutic resistance, and relapse. The intricate crosstalk between CSCs and the TME not only supports tumor sustenance but also protects CSCs from conventional therapeutic approaches. Moreover, the exploration of dormant CSCs has shed light on their role as silent drivers of recurrence, capable of evading standard treatments and reinitiating tumor growth, underscoring the urgent need for therapeutic strategies that can effectively target this subpopulation.

This review also emphasizes the pivotal role of CSCs in metastasis and recurrence, linking their plasticity and adaptability to their ability to survive hostile environments and drive systemic disease spread. Strategies for targeting CSCs, including novel therapeutic agents, pathway inhibitors, and immunotherapeutic approaches, demonstrate significant progress, as evidenced by clinical trials focusing on CSC-directed therapies. However, the outcomes underscore the inherent challenges, such as tumor heterogeneity, off-target effects, and CSC plasticity, that complicate effective treatment.

Emerging technologies, including CRISPR/Cas9 gene editing, single-cell sequencing, and high-throughput screening methods, are transforming the landscape of CSC research. These tools enable the dissection of CSC heterogeneity, providing an unprecedented resolution of their molecular profiles and facilitating the identification of novel biomarkers and therapeutic targets. However, as these technologies advance, the ethical challenges surrounding their application in CSC research and therapy development must be carefully navigated.

Looking ahead, a future landscape of CSC-targeted therapies necessitates a multi-disciplinary approach integrating basic science, clinical research, and translational strategies. The key areas for progress include the development of more specific and potent CSC-targeted agents, the refinement of drug delivery systems, and the identification of biomarkers for patient stratification in personalized medicine. Furthermore, fostering collaborations across research institutions, leveraging advanced bioinformatics platforms, and conducting robust preclinical and clinical studies are vital to bridging the gap between laboratory findings and clinical applications.

In conclusion, CSCs remain a compelling target for improving cancer treatment outcomes. By addressing the challenges of tumor heterogeneity, therapeutic resistance, and disease recurrence, future advancements in CSC research have the potential to revolutionize oncology, offering hope for more effective and durable cancer therapies.

## Figures and Tables

**Table 1:** Comparison of key characteristics between cancer stem cells (CSCs) and regular cancer cells.

Characteristic	Cancer Stem Cells (CSCs)	Regular Cancer Cells
Self-renewal	CSCs can self-renew, maintaining their population over time. They divide symmetrically to produce more CSCs or a CSC and a non-CSC.	Regular cancer cells do not exhibit the same self-renewal ability and have limited proliferative potential.
Differentiation	CSCs can differentiate into multiple cell types within the tumor, contributing to cellular diversity.	Regular cancer cells have limited differentiation potential and often form a homogeneous population.
Transdifferentiation	CSCs can undergo transdifferentiation, converting into different cell types (e.g., endothelial cells for angiogenesis), promoting tumor growth.	Regular cancer cells typically do not exhibit transdifferentiation or the ability to form specialized cell types.
Resistance to Chemotherapy	CSCs are highly resistant to chemotherapy, employing mechanisms like DNA repair, drug efflux pumps, and cell cycle arrest.	Regular cancer cells are more susceptible to chemotherapy, particularly during the proliferative phases of the cell cycle.
Role in Tumor Formation	CSCs are responsible for initiating and sustaining tumor growth, driving tumor progression and metastasis.	Regular cancer cells contribute to tumor mass but are less involved in tumor initiation and persistence.
Involvement in Tumor Progression	CSCs drive tumor progression and recurrence, even after chemotherapy, due to their ability to survive treatments.	Regular cancer cells are more likely to be eliminated by standard therapies and do not contribute to tumor recurrence as effectively as CSCs.

**Table 2:** Molecular markers and signaling pathways defining cancer stem cells (CSCs) across different cancers.

Cancer Type	Molecular Markers	Key Signaling Pathways Involved	Role of Pathways in CSC Biology
Breast Cancer	CD44+/CD24−/low, ALDH+	Wnt/β-catenin, Notch, Hedgehog	Self-renewal, differentiation, and resistance to therapies
Melanoma	CD34+CD38−	Notch, Wnt/β-catenin	Tumor initiation, progression, and metastasis
Glioblastoma	CD133	Notch, Hedgehog, Wnt/β-catenin	Self-renewal, differentiation into endothelial cells, tumor relapse
Colorectal Cancer	CD44+	Wnt/β-catenin	Self-renewal and expansion of CSCs
Pancreatic Cancer	CD133, CD44+	Notch, Hedgehog	Tumorigenesis and differentiation potential
Leukemia	CD34+	Hedgehog	Maintenance of CSC properties and chemoresistance

**Table 3:** Techniques for isolating and studying cancer stem cells (CSCs) in laboratory and clinical settings.

Technique	Short Principle	Advantages	Limitations	Application	References
Flow Cytometry and Cell Sorting	Uses fluororeacted antibodies to bind to specific CSC surface markers, enabling the isolation of CSCs from the bulk tumor population.	Highly specific and quantitative; allows for precise identification and separation of CSCs.	Limited to known CSC markers; requires expensive equipment and specialized expertise.	Isolation and analysis of CSCs for further studies.	[43,52]
Sphere Formation Assay	Measures CSCs’ ability to grow in non-adherent, serum-free conditions, forming spheroids. This assesses self-renewal and enriches CSC populations for further analysis.	Reflects CSC self-renewal ability; easy to perform and provides a functional readout of CSC activity.	Does not perfectly mimic the in vivo environment; can be influenced by culture conditions.	Studying CSC proliferation, self-renewal, and enrichment for further analysis.	[5,53]
Immunohistochemistry (IHC)	Uses antibodies to detect CSC markers in tissue samples, providing location and quantity of CSCs within the tumor.	Visualizes CSCs in tissue context, offering spatial information on their distribution.	Limited by the availability of specific antibodies; requires proper tissue preservation and preparation.	Localization and quantification of CSCs in tumor tissues.	[45]
Immunofluorescence (IF)	Fluorescently labeled antibodies bind to CSC-specific markers to visualize their presence and distribution in tissue or cultured cells.	Provides high-resolution images and helps in co-localization studies of multiple markers.	Requires specialized equipment (fluorescent microscopes); potential for signal overlap in multiplex assays.	Study of CSC marker expression and distribution in cells and tissues.	[54,55]
CSC Transplantation in Immunocompromised Mice	Involves transplanting CSCs into immunocompromised mice to observe tumorigenic potential by their ability to generate tumors.	Directly tests the tumorigenic potential of CSCs, considered the “gold standard” for evaluating their cancer-initiating capacity.	Ethical concerns; long duration; requires appropriate animal models and conditions.	Evaluating the tumorigenic potential of CSCs.	[56,57]
RNA Sequencing (RNA-seq)	Uses next-generation sequencing to analyze the transcriptome of CSCs, identifying unique gene expression profiles.	High throughput; provides comprehensive gene expression data; helps in understanding CSC molecular characteristics.	Expensive; requires bioinformatics expertise for data analysis; large amount of tissue may be required.	Identifying gene expression profiles and pathways specific to CSCs.	[58]
Proteomics	Analyzes protein expression profiles of CSCs to understand their functional roles and molecular pathways.	Provides a detailed view of the proteome; helps identify biomarkers and therapeutic targets.	Complex data interpretation; requires extensive data analysis; high cost.	Identifying proteins and pathways associated with CSC functions.	[59]
CRISPR-Cas9	Gene-editing tool that enables targeted mutations in CSCs to study the role of specific genes in CSC proliferation and differentiation.	Allows precise modification of genes in CSCs; can be used to identify essential genes and pathways for CSC functions.	Requires optimization for CSC culture; off-target effects; technical expertise required.	Gene functional studies to identify key genes involved in CSC self-renewal and differentiation.	[60]
RNA Interference (RNAi)	Uses small RNA molecules to suppress the expression of target genes in CSCs, helping identify essential pathways.	High specificity for gene silencing; enables functional analysis of specific genes.	Off-target effects; transient gene knockdown; challenges with delivery into CSCs.	Studying gene function in CSCs and identifying potential therapeutic targets.	[61,62]

**Table 4:** Research findings on the dynamic interplay between cancer stem cells (CSCs) and the tumor microenvironment (TME).

Study/Research Article	Key Findings	Implications
[68,81]	The TME plays a pivotal role in the growth, progression, spread, and resistance to treatment in cancer.	The TME shields CSCs, allowing for their survival, contributing to cancer growth and metastasis.
[69,82]	The TME consists of various cell types, such as immune cells, fibroblasts, endothelial cells, and extracellular matrix components, that create a supportive environment for CSCs.	CSCs thrive in this environment, receiving signals that enable their survival, self-renewal, and differentiation.
[70,83]	CSCs communicate with the TME through signaling pathways, cytokines, and growth factors, regulating their own proliferation, differentiation, and death.	This intercommunication allows CSCs to maintain their stemness properties and resist therapeutic interventions.
[71,84]	CSCs release factors that recruit and differentiate stromal cells and immune cells to promote a supportive TME.	The recruitment of stromal and immune cells enhances the growth and survival of CSCs, aiding in tumor progression.
[72,85]	Hypoxic conditions in tumors, caused by inadequate vascularization, influence the CSC-TME relationship.	Hypoxia in tumors leads to altered metabolic and signaling pathways that support CSC survival.
[73,86]	Hypoxia-inducible factors (HIFs) are overexpressed in CSCs in low oxygen conditions, enhancing stemness characteristics, angiogenesis, and metabolic adaptation.	HIF overexpression in CSCs promotes their ability to survive and adapt in the harsh tumor microenvironment.
[74,87]	Hypoxia regulates critical CSC signaling pathways (Wnt and Notch), inducing epithelial-to-mesenchymal transition (EMT) and increasing their invasiveness and resistance to therapies.	EMT enables CSCs to become more invasive, increasing their potential for metastasis and resistance to treatment.
[75,88]	The interaction between CSCs and the TME enhances CSC survival and contributes to tumor progression.	This relationship is critical for tumor growth and metastasis, making it a target for therapeutic strategies.
[76,83]	The TME helps CSCs evade immune system detection through cytokine secretion and the expression of co-inhibitory signals.	Immune escape mechanisms in CSCs allow them to survive and evade immune surveillance, promoting disease persistence.
[77,89]	CSCs alter the immune microenvironment by secreting immunosuppressive cytokines or by inhibiting immune responses through co-inhibitory signals.	These immune-modulating actions enable CSCs to resist immune attacks, which complicates cancer treatment.
[32,78,79,90]	CSCs and the TME play a significant role in metastasis.	The interaction of CSCs with the TME facilitates metastasis, as they can colonize distant organs and form secondary tumors.
[80,91]	CSCs exposed to stimuli from the TME undergo epithelial-to-mesenchymal transition (EMT), enabling them to move and invade other tissues.	EMT is essential for CSCs to metastasize, facilitating the spread of cancer to other parts of the body.
General Understanding	CSCs and TME are interdependent; TME supports CSCs’ self-renewal, survival, and metastatic potential, while CSCs regulate and manipulate the TME.	The interrelationship between CSCs and TME is central to tumor development, progression, immune escape, and treatment resistance. Developing therapies that target both CSCs and their niche could lead to better therapeutic outcomes and tumor control.

**Table 5:** Overview of research on cancer stem cells (CSCs) in metastasis and recurrence.

Research Article	Focus	Outcome/Findings	Significance
[14,101]	CSCs in Metastasis	CSCs are essential in metastasis, facilitating the migration of cancer cells from the primary tumor.	Highlighted the crucial role of CSCs in cancer metastasis.
[92,102]	CSC Migration in Metastasis	CSCs help in the migration from primary tumors by mediating EMT (epithelial-to-mesenchymal transition).	Demonstrated that CSCs enhance cell migration and invasion capacities, aiding metastasis.
[93,94,103]	CSCs and EMT in Metastasis	CSCs undergo EMT, a process enhancing their invasive properties, aiding detachment, and spreading through blood and lymphatic systems.	Explained how EMT facilitates CSC survival in circulation and metastasis.
[95,96,104]	EMT Signaling Pathways	Dysregulated signaling pathways (TGF-β, Wnt/β-catenin, Notch) in CSCs mediate EMT.	Focused on how environmental and signaling factors contribute to CSC-driven metastasis.
[95,96,97,104,105]	CSC Survival in Circulation	CSCs withstand circulatory system stress and evade immune detection, leading to higher survival rates in harsh environments.	Emphasized the resilience of CSCs in hostile environments like the bloodstream.
[106,107]	CSCs and Micrometastasis	CSCs can undergo mesenchymal-to-epithelial transition in distant sites to form micrometastases.	Showed the process by which CSCs can re-establish growth in secondary sites.
[97,108]	Dormancy and Recurrence of CSCs	CSCs can remain dormant for extended periods and reactivate upon stimuli, leading to delayed recurrence.	Provided insight into how CSCs contribute to delayed cancer recurrence.
[18,98]	CSCs in Pre-Metastatic Niche Formation	CSCs help form pre-metastatic niches in target organs, modulating the tissue environment for future tumor growth.	Highlighted CSCs’ role in preparing distant tissues for metastatic growth.
[99,109]	Exosomes from Renal CSCs	Exosomes from CD105+ renal CSCs enhance angiogenesis and kidney cancer metastasis to lungs by increasing VEGF and MMP-2 in lung endothelial cells.	Showed how CSC-derived exosomes can influence angiogenesis and metastasis.
[100,110]	Exosomes in Melanoma Metastasis	Exosomes from metastatic melanoma enhance metastasis by stimulating bone marrow progenitors and forming pre-metastatic niches.	Demonstrated the metastatic role of CSC-derived exosomes in melanoma.
General Observation	Role of CSCs in Metastasis and Recurrence	CSCs drive metastasis via EMT, survive harsh conditions, and establish micrometastases. CSCs contribute to recurrence through prolonged dormancy and reactivation.	Emphasized the critical role of CSCs in metastasis and cancer recurrence, with a need for targeted therapies.

**Table 6:** Role of cancer stem cells (CSCs) in tumor relapse and resistance to therapy.

Research Article	Key Findings	Outcomes	Conclusions
Study on CSC quiescence in therapy resistance [111,113,114]	CSCs remain in a quiescent state after treatment, enabling them to evade therapeutic stress	CSCs survive therapy and are capable of reactivating, leading to tumor relapse	Tumor relapse occurs due to CSCs entering a dormant state and evading treatment; new strategies are needed to target these cells
CSC DNA repair and drug efflux mechanisms [31,112]	CSCs have superior DNA repair capabilities and higher levels of drug efflux pumps	CSCs are less susceptible to DNA damage from chemotherapy, leading to drug resistance	The enhanced repair capacity and drug pump activity make CSCs resistant to conventional therapies
Mechanisms of CSC self-renewal and tumor recurrence [16,113]	CSCs can self-renew post-treatment, leading to genetic and epigenetic alterations	Genetic and epigenetic changes enable CSCs to form a new resistant tumor	CSCs contribute to tumor recurrence through self-renewal and the evolution of resistant and aggressive clones
Role of CSCs in TME regulation and angiogenesis [113,115]	CSCs regulate the tumor microenvironment (TME) and promote angiogenesis	CSCs contribute to tumor recurrence by influencing the TME and stimulating angiogenesis	Targeting CSC regulation of the TME and angiogenesis may offer new treatment approaches to prevent relapse
Targeted therapies for CSCs [113,116]	CSC-targeted therapies, including sensitization to conventional drugs and immunotherapies	Potential therapies can target CSCs and reverse their resistance to conventional treatments	New therapies targeting CSC pathways and their microenvironment may be effective in overcoming resistance and preventing relapse

**Table 7:** Exploration of dormant cancer stem cells (CSCs) and their impact on disease recurrence.

Research Article	Key Findings	Outcomes	Conclusions
Study on quiescence of dormant CSCs [117,122,123]	Dormant CSCs are in a quiescent state with reduced mitotic and oxidative activities	Dormant CSCs evade conventional therapies by staying in a dormant state, unaffected by treatments targeting dividing cells	Dormant CSCs contribute to disease recurrence by surviving treatment, underscoring the need for strategies targeting this quiescent state
CSC response to therapeutic pressure [118,124]	Dormant CSCs can survive treatment and remain latent until therapeutic pressure is relieved	When therapeutic pressure is removed, dormant CSCs reactivate and lead to tumor relapse	Disease relapse occurs due to the reactivation of dormant CSCs, which can regenerate tumors that are more invasive and resistant to prior therapies
Genetic and epigenetic alterations in dormant CSCs [119,125]	Dormant CSCs may undergo genetic and epigenetic changes during their latent period	These alterations contribute to more aggressive and therapy-resistant tumor recurrence	Genetic and epigenetic changes in dormant CSCs lead to more aggressive disease upon reactivation, necessitating new therapeutic approaches
Role of TME factors in regulating CSC dormancy [120,126]	Factors such as hypoxia, starvation, and interactions with other cells in the TME regulate CSC quiescence	Dormant CSCs are difficult to predict and target due to the influence of the TME on their state	The TME plays a crucial role in maintaining CSC dormancy, and understanding these interactions is key to preventing relapse
Targeting dormant CSCs for therapeutic strategies [117,121]	Strategies to target dormant CSCs include disrupting their survival pathways, altering the tumor niche, and enhancing immune responses	These approaches aim to eradicate dormant CSCs or prevent their reactivation	New therapeutic strategies targeting dormant CSCs, their survival pathways, and the tumor environment may enable permanent remission and improved outcomes

**Table 8:** Strategies for targeting cancer stem cells (CSCs) and overcoming challenges in cancer therapy.

Research Article	Key Findings	Outcomes	Conclusions
Monoclonal antibody targeting CD44 [127,147]	Monoclonal antibody against CD44 reduced acute myeloid leukemia in vivo by over 50%	Targeting CD44, a marker for CSCs, reduces tumor burden in vivo, highlighting its potential for CSC-targeted therapy	CD44-targeted therapy shows promise in reducing CSCs and limiting tumor progression
Cyclopamine inhibition of Hh pathway in glioblastoma stem cells [128]	Cyclopamine blocks the Hh pathway in glioblastoma stem cells, reducing growth by 40–60%	Reduction in growth of glioma cell lines with high Gli1 expression, and no neurosphere formation	Targeting the Hh pathway in glioblastoma stem cells effectively inhibits growth, presenting a viable CSC-targeting strategy
Differentiation therapy for CSCs [129]	Differentiation therapy aims to induce CSC differentiation into non-tumorigenic cells	Reduces CSC pool and enhances sensitivity to conventional therapies	Differentiation therapy is a novel approach to depleting CSCs and improving tumor response to treatment
Immunotherapy targeting CSCs [130]	Immunotherapies, including CAR-T cell therapy and CSC vaccination, trigger immune response against CSC markers	Increased immune response against CSCs, leading to tumor reduction	Immunotherapy holds promise for overcoming tumor suppressive environments and targeting CSCs
Targeting the CSC niche in the tumor microenvironment [56,133]	Strategies include inhibiting angiogenesis, targeting stromal cells, and altering extracellular matrix	Prevents CSC mobility and limits nutrient supply, reducing CSC survival	Modulating the CSC niche in the tumor microenvironment presents an effective way to target CSCs while preserving normal tissue function
Combination therapies with CSC-targeted drugs [132,134,135]	Combining CSC-targeted drugs with chemotherapy or radiation therapy enhances efficacy	Targeting both CSCs and bulk tumor cells reduces recurrence and progression	Combination therapies that target CSCs alongside conventional treatments may prevent tumor relapse and progression
RNAi and CRISPR/Cas9 for gene modification [50,51,86,136]	RNAi and CRISPR/Cas9 used to knock down or modify genes essential for CSC survival	Vulnerability of CSCs to treatment is enhanced, leading to improved outcomes	Gene modification technologies such as RNAi and CRISPR/Cas9 are valuable tools for making CSCs more susceptible to therapy
Nanoparticles targeting CSC-specific markers [137,138]	Nanoparticles can be designed to target CSC-specific markers and enhance tumor penetration	Increased specificity and efficacy of CSC-targeted therapies	Nanotechnology holds potential for delivering drugs more effectively to CSCs, enhancing therapy precision
Personalized CSC-targeted therapies [140]	Personalized therapy tailored to CSC characteristics in specific patients	More effective and individualized treatment strategies based on CSC variability across tumors	Personalized therapies are crucial for addressing the heterogeneity of CSCs and ensuring treatment efficacy
Challenges in targeting CSCs without affecting normal stem cells [141,142]	CSCs and normal stem cells share similar properties, complicating targeted therapy	Some therapies designed for CSCs may also affect normal stem cells, causing toxicity	Identifying specific markers and pathways for CSCs, along with local delivery and temporal targeting, may minimize toxicity and enhance specificity
Strategies for overcoming challenges in CSC targeting [145,146]	Approaches include targeting CSC-specific markers, using local drug delivery, and temporal targeting	These strategies reduce the risk of toxicity to normal stem cells and improve treatment efficacy	Advances in precision medicine and drug delivery systems hold promise for developing safe and effective CSC-targeted therapies

**Table 9:** Clinical trials that focus on cancer stem cell (CSC)-targeted therapeutics.

Cancer Stem Cell (CSC)-Targeted Therapeutics/Article	Trial Phase	Cancer Type	Outcome	Limitations	Practical Implications	Summarized Abstract
BBI608 (Napabucasin) [149]	Phase III	Colorectal Cancer	Did not meet primary endpoint of overall survival improvement	High toxicity, lack of efficacy	Need for better biomarkers to select responsive patient populations	Napabucasin, an investigational CSC-targeting agent, combined with FOLFIRI, failed to improve overall survival in metastatic colorectal cancer patients compared to the use FOLFIRI alone.
GDC-0449 (Vismodegib) [150]	Phase II	Basal Cell Carcinoma	Showed significant tumor reduction in advanced cases	Development of resistance, side effects like muscle spasms	Effective in targeting Hedgehog pathway, but requires combination with other therapies to prevent resistance	Vismodegib, a Hedgehog pathway inhibitor, demonstrated significant tumor reduction in patients with advanced basal cell carcinoma, though resistance and muscle spasms were notable issues.
Olaparib (Lynparza) [151]	Phase II	Ovarian Cancer	Prolonged progression-free survival in patients with BRCA mutations	Limited to patients with specific genetic mutations	Highlights importance of genetic screening in CSC-targeted therapy	Olaparib maintenance therapy significantly prolonged progression-free survival in ovarian cancer patients with BRCA mutations, underscoring the necessity of genetic screening.
PF-04449913 (Glasdegib) [152]	Phase II	Acute Myeloid Leukemia	Improved survival in combination with low-dose cytarabine	Limited efficacy as monotherapy, high cost	Potential in combination therapy, especially in older patients	Glasdegib combined with low-dose cytarabine improved survival in elderly patients with newly diagnosed acute myeloid leukemia or high-risk myelodysplastic syndromes.
BMS-833923 (XL139) [153]	Phase I	Solid Tumors	Some tumor shrinkage observed	Early trial phase, small sample size	Encouraging results warrant further investigation in larger trials	BMS-833923, targeting the Hedgehog pathway, showed preliminary signs of tumor shrinkage in patients with advanced solid tumors, meriting further research.
Demcizumab (OMP-21M18) [154]	Phase I/II	Pancreatic Cancer	Some evidence of delayed tumor progression	Cardiotoxicity, transient efficacy	Indicates potential but requires combination with cardioprotective agents	Demcizumab, targeting DLL4, exhibited potential in delaying tumor progression in pancreatic cancer but faced cardiotoxicity challenges.
PF-03084014 (Gamma-secretase inhibitor) [155]	Phase I	Breast Cancer	Partial response in some patients	Not all patients respond, gastrointestinal toxicity	Suggests need for patient selection and combination with other treatments	The gamma-secretase inhibitor PF-03084014 induced partial responses in advanced triple-negative breast cancer patients, highlighting the need for combination strategies.
VB-111 (Ofra-Vec) [156]	Phase I/II	Glioblastoma	Extended progression-free survival	Limited overall survival benefit, side effects	Demonstrates role of anti-angiogenic approach in CSC targeting	VB-111, an anti-angiogenic virotherapy, showed extended progression-free survival in recurrent glioblastoma patients, though overall survival benefit was limited.
